# An attenuated herpesvirus vectored vaccine candidate induces T-cell responses against highly conserved porcine reproductive and respiratory syndrome virus M and NSP5 proteins that are unable to control infection

**DOI:** 10.3389/fimmu.2023.1201973

**Published:** 2023-08-03

**Authors:** Rory C. F. de Brito, Kerry Holtham, Jessica Roser, Jack E. Saunders, Yvonne Wezel, Summer Henderson, Thekla Mauch, Beatriz Sanz-Bernardo, Jean-Pierre Frossard, Matthieu Bernard, Fabian Z. X. Lean, Alejandro Nunez, Simon Gubbins, Nicolás M. Suárez, Andrew J. Davison, Michael J. Francis, Michael Huether, Hafid Benchaoui, Jeremy Salt, Veronica L. Fowler, Michael A. Jarvis, Simon P. Graham

**Affiliations:** ^1^ The Pirbright Institute, Woking, United Kingdom; ^2^ The Vaccine Group Ltd., Plymouth, United Kingdom; ^3^ Oxford Vaccine Group, Department of Paediatrics, University of Oxford, Oxford, United Kingdom; ^4^ ECO Animal Health, London, United Kingdom; ^5^ Virology Department, Animal and Plant Health Agency, Addlestone, United Kingdom; ^6^ Pathology and Animal Sciences Department, Animal and Plant Health Agency, Addlestone, United Kingdom; ^7^ MRC-University of Glasgow Centre for Virus Research, Glasgow, United Kingdom; ^8^ BioVacc Consulting Ltd., Amersham, United Kingdom; ^9^ School of Biomedical Sciences, University of Plymouth, Plymouth, United Kingdom

**Keywords:** porcine reproductive and respiratory syndrome virus, T cell, vaccine, bovine herpesvirus 4, immunogenicity, protective efficacy

## Abstract

Porcine reproductive and respiratory syndrome virus (PRRSV) remains a leading cause of economic loss in pig farming worldwide. Existing commercial vaccines, all based on modified live or inactivated PRRSV, fail to provide effective immunity against the highly diverse circulating strains of both PRRSV-1 and PRRSV-2. Therefore, there is an urgent need to develop more effective and broadly active PRRSV vaccines. In the absence of neutralizing antibodies, T cells are thought to play a central role in controlling PRRSV infection. Herpesvirus-based vectors are novel vaccine platforms capable of inducing high levels of T cells against encoded heterologous antigens. Therefore, the aim of this study was to assess the immunogenicity and efficacy of an attenuated herpesvirus-based vector (bovine herpesvirus-4; BoHV-4) expressing a fusion protein comprising two well-characterized PRRSV-1 T-cell antigens (M and NSP5). Prime-boost immunization of pigs with BoHV-4 expressing the M and NSP5 fusion protein (vector designated BoHV-4-M-NSP5) induced strong IFN-γ responses, as assessed by ELISpot assays of peripheral blood mononuclear cells (PBMC) stimulated with a pool of peptides representing PRRSV-1 M and NSP5. The responses were closely mirrored by spontaneous IFN-γ release from unstimulated cells, albeit at lower levels. A lower frequency of M and NSP5 specific IFN-γ responding cells was induced following a single dose of BoHV-4-M-NSP5 vector. Restimulation using M and NSP5 peptides from PRRSV-2 demonstrated a high level of cross-reactivity. Vaccination with BoHV-4-M-NSP5 did not affect viral loads in either the blood or lungs following challenge with the two heterologous PRRSV-1 strains. However, the BoHV-4-M-NSP5 prime-boost vaccination showed a marked trend toward reduced lung pathology following PRRSV-1 challenge. The limited effect of T cells on PRRSV-1 viral load was further examined by analyzing local and circulating T-cell responses using intracellular cytokine staining and proliferation assays. The results from this study suggest that vaccine-primed T-cell responses may have helped in the control of PRRSV-1 associated tissue damage, but had a minimal, if any, effect on controlling PRRSV-1 viral loads. Together, these results indicate that future efforts to develop effective PRRSV vaccines should focus on achieving a balanced T-cell and antibody response.

## Introduction

Porcine reproductive and respiratory syndrome (PRRS) is a highly infectious disease that causes major economic losses in the pig industry worldwide (1–4). The causative agents, PRRSV-1 (*Betaarterivirus suid 1*) and PRRSV-2 (*Betaarterivirus suid 2*), are single-stranded positive-sense RNA viruses in the *Arteriviridae* family of the order *Nidovirales* (5, 6). PRRSV infection can lead to reproductive failure in sows and respiratory disorders in pigs of all ages, with animals showing an increased susceptibility to secondary viral and bacterial infections (1, 7). Both PRRSV-1 and -2 are rapidly evolving and display high genetic variation (8, 9). Heightened pathogenic strains of both viral species have emerged previously, and such emergence remains a threat (10–12). The antigenic heterogeneity of PRRSV is a major challenge in disease control strategies using immunization with the currently available inactivated PRRSV or modified live virus (MLV) vaccines (13, 14). PRRSV MLV vaccines have been favored over inactivated vaccines because they are more immunogenic and confer higher levels of protection (14, 15). However, MLV vaccines have safety concerns regarding virulence reversion, offer only limited protection against heterologous PRRSV strains, and do not prevent viral shedding (16–18). Therefore, there remains a considerable need for safe PRRSV vaccines that can induce broad levels of protection and provide more effective PRRSV control.

Several experimental PRRSV vaccines have been developed using a variety of platforms, including viral vectors and subunit vaccines (19). However, vaccine design is complicated by a lack of clarity regarding the relative role of antibodies compared to T cells in the control of PRRSV replication and associated diseases. Pigs mount an early antibody response after PRRSV infection but then show a delayed generation of neutralizing antibodies, which are typically restricted in breadth and protective capacity (20–23). Target antigens for virus vector/subunit vaccines have predominantly been those containing epitopes recognized by neutralizing antibodies, namely envelope glycoproteins GP2, GP3, GP4, and GP5, many of which target GP5 together with M, with which GP5 forms a heterodimeric complex (22, 24–27). These neutralizing antibody-focused vaccines display varying levels of immunogenicity, with protection typically only partial (27–30). Cellular responses, primarily involving IFN-γ secreting T cells, are considered important for viral clearance (31–33). PRRSV-specific IFN-γ secretion occurs from both CD4^+^ and CD8^+^ T cells, with one study showing CD8^+^ cells as the predominant T-cell subset infiltrating the lungs of PRRSV-2 infected pigs and CD4^+^ T helper cells in the blood and lymphoid tissues, coinciding with a reduction in viremia (34–38). IFN-γ has been shown to reduce PRRSV infection in macrophages *in vitro*, and IFN-γ responses have been associated with a more effective clearance of some PRRSV-1 strains *in vivo* (39–42). PRRSV proteins containing T-cell epitopes are being increasingly explored as antigenic targets for inclusion in PRRSV vaccines (43, 44). Multiple studies have identified T-cell epitopes in structural proteins, such as GP5 and M proteins, as well as in non-structural proteins (NSPs), including NSP2, NSP5, and NSP9 (45–49). The development of vaccine candidates targeting more highly conserved, non-GP-based antigens as T-cell targets is also an area of current interest based on their potential to achieve broad cellular responses, overcoming existing vaccine limitations related to PRRSV variability (50, 51).

Herpesviruses naturally elicit effector-memory T-cell-mediated immunity and this feature has been utilized in attempts to generate herpes-based viral vectors that enhance CD8 T-cell responses (52–55). Examples of such vectors include bovine herpesvirus 4 (BoHV-4) and cytomegalovirus (CMV) (56, 57), which are attractive recombinant viral vectors because of their ease of genomic manipulation, ability to accommodate and express large antigenic inserts, and the ability of vectors to infect various host cell types (58–60). For instance, recombinant BoHV-4 has been used as a promising vector for several experimental vaccines, including Nipah, Peste des petits ruminants, and Ebola viruses (61–65). Therefore, this study aimed to exploit the natural potential of herpesviral vectors to enhance cell-mediated immunity and test the vaccine potential of BoHV-4 vectored delivery of conserved PRRSV-1 antigens. PRRSV-1 M and NSP5 were selected as T-cell antigens because proteome-wide peptide library screening identified these as major antigens containing CD4^+^ and CD8^+^ T-cell epitopes conserved across multiple divergent PRRSV strains and recognized in outbred pigs (45, 51). Analysis of lungs during the resolution of PRRSV-1 infection also showed substantial M and NSP5 specific CD8^+^ T-cell responses at this effector tissue site (50). Therefore, PRRSV M and NS5 proteins represent ideal target antigens for inclusion in a BoHV-4 vectored vaccine candidate.

## Material and methods

### Cell culture

Porcine bronchoalveolar lavage cells (BALC) and peripheral blood mononuclear cells (PBMC) were cultured in RPMI-1640 medium (cRPMI) supplemented with 10% fetal bovine serum (FBS), 100 IU/mL penicillin, and 100 µg/mL streptomycin (all reagents from Thermo Fisher Scientific, Loughborough, UK). Meat Animal Research Center-145 (MARC-145) and Madin-Darby bovine kidney (MDBK) cells (CCL-22, Manassas, USA) were cultured in Dulbecco’s modified Eagle’s medium (DMEM; Thermo Fisher Scientific), supplemented as described above. All the cells were cultured at 37°C with 5% CO_2_ in a humidified incubator.

### Construction of recombinant BoHV-4

A synthetic M and NSP5 fusion construct based on the PRRSV-1 Olot/91 strain sequence (GenBank accession no. KC862570) was designed and codon-optimized for expression in pigs (*Sus scrofa*). The M-NSP5 fusion uses a synthetic helical linker (66) to link full-length M and NSP5 followed by the addition of a V5 epitope at the carboxyl terminus. The synthesized fusion gene (GeneArt; Thermo Fisher Scientific) was cloned into a shuttle vector using standard cloning procedures to place expression under the control of the human cytomegalovirus (hCMV) major immediate-early promoter. The shuttle vector also contained an FLP recombinase recognition target (FRT)-flanked kanamycin-resistance (KanR) gene for the selection of recombinants. All restriction enzymes used for cloning were purchased from New England Biolabs (Hitchin, UK) and were used as according to the manufacturer’s instructions. The BoHV-4-M-NSP5 bacterial artificial chromosome (BAC) was obtained by E/T recombination of the shuttle vector into the V. test wild-type (WT) BoHV-4 BAC (67) within EL250 recombinogenic bacteria (kindly provided by Donald Court, National Cancer Institute), thereby replacing the BoHV-4 gene ORF73 (67). Bacterial clones containing recombinant BACs were selected for kanamycin resistance followed by removal of the KanR marker by arabinose induction of FLP recombinase within the bacteria. Kanamycin-sensitive bacterial colonies containing recombinant BACs were screened by restriction fragment length polymorphism to confirm the removal of the KanR marker and the absence of gross genomic rearrangements. PCR using primers flanking ORF73 was used to confirm the correct insertion of the transgene within the BoHV-4 genome, and direct DNA Sanger sequencing was used to confirm the integrity of the M-NSP5 expression cassette. Whole-genome next-generation sequencing (NGS) was used to confirm the absence of any unanticipated genetic alterations within the remainder of the BoHV-4-M-NSP5 BAC (68).

For virus reconstitution, BoHV-4-M-NSP5 or BoHV-4 wild-type (WT) BACs were transfected using standard methods into embryonic bovine lung cells (EBL-Cre) (67) —EBL-Cre cells expressing Cre recombinase enabling excision of the floxed BAC cassette within the BoHV-4 BAC. For viral characterization, viral DNA was extracted from infected cells using a QIAamp MinElute Virus Spin Kit (QIAGEN, Manchester, UK). PCR and Sanger sequencing were used to confirm transgene integrity (for BoHV-4-M-NSP5), and NGS was used to confirm the integrity of the entire BoHV-4/M-NSP5 or BoHV-4 WT virus genome. Expression of the V5 tagged M-NSP5 fusion protein was confirmed over at least five passages by western blot analysis of BoHV-4-M-NSP5 infected MDBK cell lysates using a V5 epitope-specific monoclonal antibody (Bio-Rad Antibodies, Watford, UK) (**Supplementary Figure 1**). Prior to use in animals, virus stocks were titrated on MDBK cells and the absence of bacterial contamination was confirmed by culture. Flanking PCR confirmed the correct genome size of BoHV-4-M-NSP5 and absence of WT BoHV-4. western blotting analysis confirmed the expression of M-NSP5. Sanger sequencing and whole-genome sequencing were used to confirm the integrity of the BoHV-4-M-NSP5 virus stock.

### Challenge viruses

PRRSV-1 subtype 1 UK isolate 21301-19 was propagated in BALC. BALC propagated PRRSV-1 subtype 3 isolate LT3 was kindly provided by Dr Christine Tait-Burkhard, The Roslin Institute, University of Edinburgh, UK. Virus titers were determined in BALC, followed by calculation of the 50% median tissue culture infectious dose (TCID_50_) using the Spearman-Karber method after 96 hours incubation and immunoperoxidase staining, as described previously (69). The identity between the predicted amino acid sequences of PRRSV-1 Olot/91 and PRRSV-1 21301-19 (GenBank accession nos. OR102332and OR102331) M protein was 95.38% and NSP5 was 90.00%. The M and NSP5 sequence identity between PRRSV-1 Olot/91 and PRRSV-1 LT-3 (GenBank accession no. OR146966) was 95.38% and 90.00%, respectively.

### Synthetic peptides

Overlapping peptides were synthesized (Mimotopes Pty Ltd, Heswall, UK) using the predicted amino acid sequences of the PRRSV-1 Olot/91 strain M and NSP5 proteins (GenBank accession no. KC862570; 16-mers offset by four amino acids). In total, 81 peptides (NSP5 represented by 42 peptides; M represented by 39 peptides) were reconstituted in sterile DMSO (Sigma-Aldrich, Merck Life Science, Gillingham, UK), pooled and aliquoted. Thirty-three overlapping 20-mer peptides, offset by 10 amino acids (Mimotopes), represent the predicted M and NSP5 sequences of the PRRSV-2 16CB02 isolate (39, 70) GenBank accession nos. MZ700336.1 and OQ446603 were similarly pooled. The identity between the predicted amino acid sequences of PRRSV-1 Olot/91 and PRRSV-2 16CB01 M protein was 79.00% and NSP5 was 70.59%, respectively.

### Vaccination and challenge studies

Two animal experiments, approved by The Pirbright Institute, Animal and Plant Health Agency and Poulpharm Animal Welfare and Ethics Committees, were conducted in accordance with the UK Animals (Scientific Procedures) Act 1986 (Project License P6F09D691) (Study 1) and the European Union Directive 2010/63/EU (Study 2). Both studies utilized PRRSV-naïve (antibody- and virus-free), weaned piglets sourced from commercial, high-health status farms. The animals were confirmed by PCR to be free from influenza A virus, porcine parvovirus, porcine circovirus 2, and porcine circovirus 3. Studies were performed following the principles of Good Clinical Practice (GCP; VICH GL 9, European Medicines Agency) and included negative control groups. Studies were partially masked, i.e., blinded to the statistician and study/laboratory personnel responsible for recording or assessing clinical, pathological, virological, and immunological data. Block randomization (Matlab (version R2020b), The MathWorks Inc.) by weight, litter, and sex (the latter for Study 2 only) was used to allocate pigs to groups, including euthanasia time points. In Study 1, the treatment groups were housed in separate pens/rooms, with no direct contact between the groups. In Study 2, the treatment groups were housed in separate pens/rooms until the challenge to prevent cross-contamination between groups. On day 39, the pens were randomly allocated to one of three rooms in a manner in which each room had one pen from each of the treatment groups. The animals were provided with water *ad libitum* and fed twice daily on a commercial diet.

### Study design

#### Study 1

Thirty-six, 6-week-old, Large White × Landrace × Hampshire crossbred female piglets were assigned to a mock-vaccinated negative control group (A) and two vaccinated groups (B and C) (**Table 1**). Four sentinel pigs, either vaccinated or challenged, and euthanized on day 42 (D42) were co-housed with groups B and C (two pigs/group) to assess the shed and spread of the recombinant vector. Within each treatment group, six pigs were randomly allocated for euthanization on D49 or D70. Group sizes were calculated according to the efficacy criteria of PRRSV-1 RNA (Cq) levels and lung lesion scores. In an earlier unpublished experiment using the PRRSV-1 21301/19 challenge strain, the standard deviation (SD) in qRT-PCR cycle quantification (Cq) values for PRRSV-1 RNA in serum between animals was approximately 2. Therefore, based on observed differences in Cq values between vaccinated and unvaccinated pigs, a group size of six pigs would detect a difference between groups with 80% power and 95% confidence (power.t.test function in R (version 4.0.5) (71)). Consequently, a group size of six pigs was predicted to be sufficient to demonstrate that vaccination had an impact on PRRSV-1 dynamics in pigs. In the same study, the mean gross lung lesion scores in three vaccinated and three unvaccinated pigs were 2.7 and 15.7, respectively, with a pooled SD of 5.4. Assuming a similar pattern of differences in the current study, a group size of six was predicted to be able to detect a difference in each gross lung lesion score with 95% power and 95% confidence (power.t.test function in R (version 4.0.5) (71)).

**Table 1:** Study 1 immunization groups and schedule.

Group	Days post-immunization				
	0	21	42	49	70
**A** (Negative control; n = 12)	WT BoHV-4 vector 10^9^ pfu (i.m.)	WT BoHV-4 vector 10^9^ pfu (i.m.)	10^5^ TCID_50_ PRRSV-1 strain 21301/19 (i.n.)	Euthanasia, post-mortem examination, and sample collection (n = 6)	Euthanasia, post-mortem examination, and sample collection (n = 6)
**B** (Experimental vaccine, prime only; n = 12)*	BoHV-4-M-NSP5 10^9^ pfu (i.m.)	Placebo (DMEM) (i.m.)		Euthanasia, post-mortem examination, and sample collection (n = 5)	Euthanasia, post-mortem examination, and sample collection (n = 6)
**C** (Experimental vaccine, prime-boost; n = 12)*	BoHV-4-M-NSP5 10^9^ pfu (i.m.)	BoHV-4-M-NSP5 10^9^ pfu (i.m.)		Euthanasia, post-mortem examination, and sample collection (n = 5)	Euthanasia, post-mortem examination, and sample collection (n = 6)

*Four unvaccinated sentinel animals were co-housed with groups B and C (two/group) and were euthanized on D42.

##### Vaccination

Animals were inoculated with 1 × 10^9^ plaque-forming units (pfu) of WT BoHV-4 vector (group A) or BoHV-4-M-NSP5 (groups B and C) diluted in 2 mL DMEM via intramuscular (i.m.) injection into the brachiocephalic muscle on D0. On D21, pigs in groups A and C received a second identical inoculation, whereas pigs in group B received inoculation with 2 mL DMEM.

##### Challenge

Animals were challenged intranasally (i.n.) on D42 with 1 × 10^5^ TCID_50_/mL PRRSV-1 strain 21301/19 diluted to 2 mL in Dulbecco’s PBS without calcium and magnesium (DPBS; 1 mL/nostril), using a mucosal atomization device (MAD 300, Wolfe Tory Medical, Salt Lake City, USA). Back titrations of both the vaccine and challenge viruses were performed as described above to confirm the doses administered. Six pigs (unless stated otherwise) from groups A to C were euthanized on D49 and D70. Animals were sedated by i.m. injection with a cocktail of Domitor (Medetomidine—1 mg/mL) and Zoletil (Tiletamine and Zolazepam—50 mg/mL) at a concentration of 0.5 mL/10 kg body weight, before an overdose of pentobarbital sodium anesthetic (Pentoject—20 mL/animal) by intravenous injection in the marginal ear vein, followed by exsanguination, to enable post-mortem examination of lungs and collection of tissue samples. One pig was euthanized (D28) and another died (D42) during the study because of vaccine-unrelated complications, resulting in only five pigs from groups B and C being euthanized on D49.

#### Study 2

Thirty-six, 5–6-week-old, Hypor × Germain Pietrain crossbred, mixed-sex piglets were assigned to a mock vaccinated negative control group (A), an MLV-vaccinated positive control group (B), and a BoHV-4-M-NSP5 vaccinated group (C) (**Table 2**). One sentinel pig, neither vaccinated nor challenged and euthanized on D39, was co-housed with the negative control group (A). Within each treatment group, six pigs were randomly allocated to euthanize on D52 or D53. Group sizes were based on previous experiments with the same PRRSV-1 challenge strain: the SD in Cq values for viremia was 1.34, while for lung pathology score, SD was 1.6. A group size of 12 pigs was predicted to be able detect a difference between groups in Cq values of 3.32 (approximately equivalent to a one log_10_ difference in virus titer) with >99% power and 95% confidence (power.t.test command in R (version 4.0.5) (71)). Similarly, a group size of 12 pigs was predicted to be able to detect a 50% reduction in the lung pathology score with 83% power and 95% confidence. These differences in Cq and lung pathology scores were predicted to be sufficient to show that the test vaccines have a biologically meaningful effect on viremia and pathology compared with unvaccinated pigs.

**Table 2:** Study 2 immunization groups and schedule.

Group	Days post-immunization				
	0	21	42	52	53
**A** (Negative control; n = 12) *	DMEM (i.m.)	DMEM (i.m.)	10^6^ TCID_50_ PRRSV-1 strain LT3 (i.n.)	Euthanasia, post-mortem examination, and sample collection (n = 6)	Euthanasia, post-mortem examination, and sample collection (n = 6)
**B** (Positive control); n = 12)	Licensed PRRSV-1 MLV vaccine (i.m.)	DMEM (i.m.)		Euthanasia, post-mortem examination, and sample collection (n = 6)	Euthanasia, post-mortem examination, and sample collection (n = 6)
**C** (Experimental vaccine; n = 12)	BoHV-4-M-NSP5 10^9^ pfu (i.m.)	BoHV-4-M-NSP5 10^9^ pfu (i.m.)		Euthanasia, post-mortem examination, and sample collection (n = 6)	Euthanasia, post-mortem examination, and sample collection (n = 6)

*One unvaccinated sentinel animal was co-housed with the negative control group and was euthanized on D39.

##### Vaccination

On D0, animals were inoculated i.m. by injection into the brachiocephalic muscle with 2 mL DMEM (group A), 1 mL Ingelvac PRRSFLEX^®^ EU (10^4.4^–10^6.6^ TCID_50_ PRRSV-1 strain 94881) as described by the manufacturer (Boehringer Ingelheim Animal Health) (group B) or 1 × 10^9^ pfu BoHV-4-M-NSP5 (group C) diluted in 2 mL DMEM. On D21, groups A and B were inoculated with DMEM, whereas group C received a second inoculation of BoHV-4-M-NSP5.

##### Challenge

Animals were challenged i.n. on D42 with 1 × 10^6^ TCID_50_/mL PRRSV-1 strain LT3 diluted to 5 mL in DPBS (2.5 mL/nostril) using a MAD 300 device. Back titrations of both the vaccine and challenge viruses were performed as described above. Six pigs per group were euthanized on D52 and D53. Pigs were first sedated by i.m. injection with a mixture of xylazine, tiletamine, and zolazepam (XylM 2% + Zoletil 100) (each at 20 mg/mL) at a concentration of 0.22 mL/kg body weight, before euthanasia by intracardial injection of an overdose of pentobarbital. Following euthanasia, the pigs were exsanguinated to facilitate lung lesion scoring.

### Clinical monitoring, pathological examination, and sample collection

Animals were clinically scored and rectal temperatures were measured daily for a week after each vaccination and for a maximum of 14 days post-challenge (dpc; **Supplementary Tables 1**, **2**). Venous blood samples were collected in BD SST and heparin vacutainers (Thermo Fisher Scientific). Nasal swabs were collected using cotton swabs (Scientific Laboratory Supplies, Nottingham, UK), which were placed into 1 mL of Medium 199 (Merck Life Science) supplemented with 25 mM HEPES, 0.035% sodium bicarbonate, 0.5% BSA, 100 IU/mL penicillin, 100 μg/mL streptomycin, and 0.25 μg/mL nystatin (all reagents from Thermo Fisher Scientific). Following euthanasia, the lungs were removed, and digital pictures of the dorsal and ventral aspect of the organ were taken. The presence of gross lesions in each pulmonary lobe was evaluated blindly by a competent veterinary pathologist and scored semi-quantitatively to estimate the percentage of the lung surface affected by pneumonia using a system adapted from Halbur et al. (72). To provide additional quantitative data on the extension of gross lesions in the lungs across the two studies, digital images of the ventral and dorsal surfaces of the lungs were analyzed using ImageJ 1.53. Briefly each lung lobe was manually delineated, and the dorsal and ventral surfaces were calculated using the software package. The areas of pneumonia in each aspect of the lobe were measured in a similar way and the percentage of the total areas with pneumonia was calculated per lobe and for the whole lung. Two representative samples from a standardized location within each of the cranial, middle, and caudal lobes of the right lung were immersed in 10% neutral buffered formalin for histological processing and scoring as previously described (70). Bronchoalveolar lavage was performed on the left lung using RPMI-1640 with 100 IU/mL penicillin, 100 µg/mL streptomycin, and 2% FBS until 100 mL of BAL fluid (BALF) was obtained. The spleen, thymus, and inguinal lymph nodes were removed and aliquots were placed in DPBS with 100 IU/mL penicillin, and 100 μg/mL streptomycin and 2% FBS for cell isolation.

### Serum and cell isolation

Serum was isolated by centrifugation of SST vacutainers at 1,300×*g* for 10 minutes (min) at room temperature and stored at −80°C. PBMC were isolated from heparinized blood diluted 1:1 in DPBS by centrifugation in a Leucosep tube (Thermo Fisher Scientific) containing Histopaque 1.077 (Merck Life Science). After centrifugation, the plasma was removed and PBMC were harvested and washed in DPBS. Contaminating erythrocytes were lysed by incubation in RBC Lysis Buffer (BioLegend, London, UK) for 5–7 min. PBMCs were washed twice with DPBS and resuspended in cRPMI for immediate use or cryopreserved in 10% DMSO (Merck Life Science) in FBS. BALF was centrifuged at 500×*g* for 10 min, and the supernatant was aliquoted and stored frozen at −80°C. The BALC were washed twice in DPBS and filtered through a 100 µm cell strainer (Thermo Fisher Scientific). The spleen, thymus, and inguinal lymph node tissues were finely chopped in DPBS and the cells were dissociated by applying pressure to the syringe barrel. Cells were then passed through a 100 µm cell strainer and washed, and erythrocytes were lysed before two final washes in DPBS. BALC and lymphoid tissue cells were resuspended in cRPMI or cryopreserved as described for PBMC.

### PRRSV RNA quantification by RT-qPCR

PRRSV-1 viremia and lung loads following challenge were assessed by RT-qPCR (73). RNA was extracted from sera and BALF using the MagMAX CORE Nucleic Acid Purification Kit and KingFisher™ Flex instrument (Thermo Fisher Scientific). Briefly, 200 μL of the sample was mixed with microbeads, lysis buffer, and binding solution according to the manufacturer’s instructions. A simple workflow was used for serum analysis, whereas a complex workflow was used for BALF analysis. Exogenous internal RNA extraction controls were also included for all samples. RNA was eluted into a 96-well plate and RT-qPCR reactions were performed using the multiplex VetMAX™ PRRSV EU & NA 2.0 Kit (Thermo Fisher Scientific). Cq values were obtained using a 7500 Fast PCR system (Applied Biosystems™, Thermo Fisher Scientific) and Cq values below 40 were considered positive.

### Quantification of BoHV-4 in nasal swabs

BoHV-4 was quantified in nasal swab fluid samples from the BoHV-4-M-NSP5 prime-boost group (Study 1) using the VetMAX™ BHV Type 4 Kit (Thermo Fisher Scientific). In brief, DNA was extracted from nasal swab fluid samples collected on D0, D1, D3, D7, D21, D22, D24, D28, and D42 using the MagMAX CORE Nucleic Acid Purification Kit and Kingfisher Flex System simple workflow. Cellular β-actin was used as a DNA extraction control and Cq values below 45 for BHV-4 were considered positive. To determine the levels of infectious BoHV-4 in PCR-positive swabs, swab fluids were titrated against MDBK cells, and cytopathic effects were scored after 10–14 days.

### IFN-γ ELISpot assay

PBMC, BALC, and lymphoid tissue cells were plated at 2.5 × 10^5^ cells/well in 96-well PVDF membrane plates (Merck Life Science) coated with anti-porcine IFN-γ mAb (clone P2G10; BD Pharmingen, Oxford, UK). Cells were either left unstimulated (cRPMI), stimulated with M-NSP5 peptide pool at 1 μg/mL, or stimulated with 5 µg/mL concanavalin A as a positive control. All experiments were performed in triplicate. The plates were incubated overnight at 37°C and 5% CO_2_. The cells were removed, and secondary biotinylated anti-porcine IFN-γ mAb (clone P2C11, BD Pharmingen) was added, followed by further washing. Plates were then developed with streptavidin–alkaline phosphatase and a BCIP/NBT colorimetric substrate (both Mabtech, 2BScientific, Kirtlington, UK). The spots were counted using an ImmunoSpot Reader (Cellular Technology Limited, Ohio, USA), and the results were expressed as the number of IFN-γ-producing cells per 10^6^ cells minus the average number of IFN-γ-producing cells in unstimulated wells.

### Intracellular cytokine staining

For analysis of intracellular cytokine production, cryopreserved PBMC and BALC from Study 1 were resuscitated and 5 × 10^5^ cells were added to the wells of 96-well round-bottom plates and stimulated in triplicate with 100 μL of either M-NSP5 peptide pool at 1 μg/mL, PRRSV-1 21301/19 at a multiplicity of infection of 0.1, cRPMI alone as a negative control, or pokeweed mitogen (5 µg/mL; Merck Life Science) as a positive control. After incubation at 37°C for 2 h, stimulated cells were incubated with 1  μg/mL GolgiPlug (BD Biosciences, Oxford, UK) for a further 12 h and then stained for surface markers using the following conjugated mAbs: CD3-PE-Cy-7 (clone BB23-8E6-8C8, SouthernBiotech, Cambridge Bioscience, Cambridge, United Kingdom), CD4α-PerCP-Cy5.5 (clone 74-12-4, BD Biosciences), CD8α-FITC (clone 76-2-11, BD Biosciences), CD8β-APC-Cy7 (clone PPT23, Bio-Rad Antibodies; conjugated using APC-Cy7 Lightening Link conjugation kit, Abcam, Cambridge, UK), and Live/Dead Fixable Aqua viability dye (Thermo Fisher Scientific). Cells were fixed and permeabilized using CytoFix/CytoPerm solution (BD Biosciences), washed in Perm/Wash buffer (BD Biosciences), and then intracellular staining incubated with the following conjugated mAbs: IFN-γ-Alexa Fluor 647 (clone CC302, BioRad Antibodies), TNF-Brilliant Violet 711 (BV711; clone MAb11, BioLegend), and perforin-Brilliant Violet 421 (BV421; clone δG9, BioLegend). Cells were then washed, resuspended in DPBS with 2% FBS (FACS buffer), and analyzed using a Cytek Aurora flow cytometer (Cytek Biosciences, Fremont, CA, USA). FlowJo v10 software (BD Biosciences) was used for the flow cytometric data analysis.

### Cell proliferation assay

PBMC were resuspended and labeled using the CellTrace™ Violet Cell Proliferation Kit (Thermo Fisher Scientific). Cells were then seeded into 96-well plates at 5 × 10^5^ cells/well and cultivated in triplicate with M-NSP5 peptides (0.5 µg/mL) or with cRPMI alone as a negative control. Following incubation at 37°C for 4 days, cells were surface stained with mAbs: CD4a-PerCP-Cy5.5 (clone 74-12-4, Southern Biotech), CD8a-FITC (clone 76-2-11, Southern Biotech), CD3-PECy-7 (BB23-8E6-8C8, Southern Biotech), CD25-AF647 (clone K231.3B2, BioRad Antibodies), and Zombie Near Infra-Red Fixable Viability Kit (BioLegend). After washing with FACS buffer, cells were fixed and permeabilized with eBioscience™ FOXP3/Transcription Factor Staining Buffer Set (Thermo Fisher Scientific), before staining with FOXP3-PE mAb (clone FJK-16s, Thermo Fisher Scientific). Cells were then washed, resuspended in FACS buffer, and proliferation was measured using a Cytek Aurora flow cytometer and subsequent analysis using FlowJo software. The relative proliferation was calculated as the percentage of proliferating cells in each gated cell population.

### BALC phenotyping

For BALC phenotyping, cryopreserved cells were resuspended, seeded into a 96-well plate with cRPMI, and stained on the same day using the antibody staining panel described above for the proliferation assay.

### IL-10 ELISA

Cell culture supernatants were collected from the PBMC proliferation assay plates at the end of the incubation period (see above) and frozen at −80°C until required. IL-10 was quantified in culture supernatants using the Porcine IL-10 DuoSet ELISA kit, as described by the manufacturer (R&D Systems, Bio-Techne, Abingdon, UK).

### Detection of PRRSV-specific antibodies in serum and BALF

Serum (D0, D21, D42, and D70) and BALF (D70) samples from Study 1 were assessed for PRRSV-specific antibodies using infected cell lysates as antigens, as previously described (70). In brief, PRRSV-1 Olot/91 infected MARC-145 cell pellets were sonicated (20 kHz, 3 × 30 s) in alkaline lysis buffer (1% Triton X-100, 50 mM borate, 150 mM NaCl, pH 9) and clarified. Nunc MaxiSorp™ plates (Thermo Fisher Scientific) were coated overnight at 4°C with 1 µg lysate/well in carbonate buffer (Sigma-Aldrich, Merck Life Science, Gillingham, UK). After blocking with 4% milk in PBS for 1 h, serum diluted to 1:50 and BALF diluted to 1:2 was added and incubated for 1 h at 37°C. Goat anti-pig IgG-Fc antibody HRP-conjugated (Bethyl Laboratories, Cambridge Bioscience, Cambridge) or Goat anti-pig IgG (H + L) antibody HRP-conjugated (Bethyl Laboratories, Cambridge Bioscience, Cambridge) was then added to serum or BALF plates, respectively. After incubation for 1 h at 37°C, tetramethylbenzidine (TMB) substrate was added, and the reaction was stopped by adding 2 M sulfuric acid. Absorbance values were measured immediately at 450 nm (OD_450_) using a GloMax^®^ Explorer Multimode Microplate Reader (Promega, Southampton, UK).

Serum (D42, D49, D56, D63, and D70) and BALF (D70) samples from Study 1 were additionally assessed using the PrioCHECK™ Porcine PRRSV Ab Strip Kit (Thermo Fisher Scientific), which detects PRRSV nucleocapsid protein-specific antibodies. All steps were performed according to the manufacturer’s instructions. Briefly, serum diluted 1:20 and BALF diluted 1:2 were incubated in pre-coated strips for 1 h at RT. The plates were washed and incubated for 1 h with an anti-swine antibody labelled with HRP. After washing, the plates were incubated with TMB substrate for 20 min, and the reaction was stopped by the addition of 2 M sulfuric acid. OD_450_ values were measured as described above. The results were expressed as the percentage of positivity (PP) according to the formula: PP = (OD_450_ sample − OD_450_ negative control/OD_450_ positive control − OD_450_ negative control) × 100.

### Detection of PRRSV neutralizing antibodies in serum

PRRSV neutralizing antibody titers were measured in serum samples (D42 and D70) from Study 1, by adaptating a previously described protocol (74). Briefly, heat-inactivated serum, serially diluted 2-fold from 1:2, was incubated with 400 TCID_50_ of PRRSV-1 Olot/91 or 21301-19 for 1 h at 37°C. MARC-145 cells (Olot/91) or porcine BALC (21301–19) were added to the serum-virus mixtures. After 2 days of incubation at 37°C and 5% CO_2_, the cells were fixed and permeabilized (2% paraformaldehyde, 0.1% Triton X-100 in PBS) for 10 min at RT and blocked with 10% goat serum in PBS. The cells were stained with an anti-PRRSV N mAb (SDOW17-A, Rural Technologies Inc., Brookings, SD, USA). MARC-145 cells were stained with goat anti-mouse IgG antibody conjugated to Alexa Fluor 488 (Thermo Fisher Scientific) and images were acquired using the Incucyte^®^ Live-Cell Analysis System (Sartorius, Royston, UK). BALC were stained with goat anti-mouse IgG (H + L) HRP-conjugated antibody (Thermo Fisher Scientific), followed by the addition of AEC substrate solution (Thermo Fisher Scientific), and the infected cells were assessed by microscopy. Neutralizing antibody titers were calculated as the reciprocal serum dilution that neutralized the infection in 50% of the wells.

### Data and statistical analysis

Data were analyzed using the GraphPad Prism 9.0.1 software. Two-way ANOVA with Geisser Greenhouse correction followed by Tukey’s test was performed (lung lesion scores, viral load, T cell, and BALC analyses after unstimulated condition correction, and antibody responses). As the distributions were not normal (Anderson–Darling test), the non-parametric Kruskal–Wallis test was used. If this was significant (P <0.05), it was followed by Dunn’s test or Wilcoxon rank-sum test for multiple comparisons to identify differences among groups. An unpaired non-parametric Mann–Whitney U test was used to compare the data between the two groups.

The rectal temperatures of pigs were used to determine the number of pigs with elevated rectal temperature and the duration of elevated temperature for three time periods: post-prime immunization (D0–D7), post-boost immunization (D21–D28), and post-challenge (D42–D56). A threshold for elevated rectal temperature was defined based on deviations from the mean rectal temperature prior to challenge (on D42) for each pig (i.e., the residuals), such that a pig’s rectal temperature was deemed to be elevated if it was above its mean prior to challenge, plus the 90th percentile of the residuals for all pigs (0.3°C). The duration of elevated rectal temperature was defined as the time between the first and last observations at an elevated temperature. Rectal temperature analysis was implemented in MATLAB (version R2020b; The MathWorks Inc.).

## Results

### Assessment of the safety of BoHV-4 vectored PRRSV-1 M and NSP5

Clinical signs were scored, and rectal temperatures were measured daily for 7 days post-prime and boost immunizations in Study 1 (**Supplementary Figure 2A**). Inoculation of pigs with BoHV-4 or BoHV-4-M-NSP5 did not induce any clinical signs other than the proportion of pigs (29%–67%) displaying an elevated rectal temperature (defined as a rectal temperature >0.3°C above the mean for each pig prior to challenge) for a short duration (<3 days). Pigs immunized with BoHV-4 or BoHV-4-M-NSP5 gained weight at an equivalent rate post-vaccination, and this continued following the PRRSV-1 challenge (**Supplementary Figure 2B**). The shedding of BoHV-4-M-NSP5 was assessed using nasal swabs from prime-boost and sentinel pigs in Study 1. Very low levels of BoHV-4 DNA were detected in swab fluids from both vaccinated and sentinel animals (**Supplementary Figure 3**), but no infectious virus was isolated from MDBK cells (data not shown).

### Assessment of the immunogenicity of BoHV-4 vectored PRRSV-1 M and NSP5

**Figure 1:**
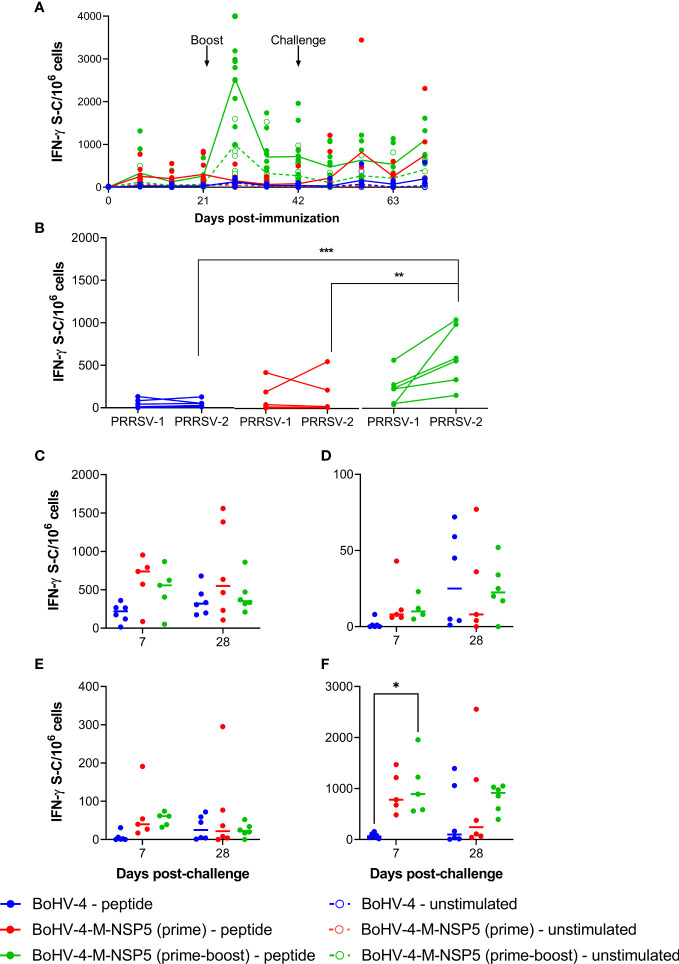
Assessment of IFN-γ response induced by BoHV-4-M-NSP5. PRRSV-1 M/NSP5 specific IFN-γ responses were assessed following one (prime) or two (prime-boost) immunizations of pigs with BoHV-4-M-NSP5, followed by challenge with PRRSV-1 21301/19 (Study 1). Control pigs were immunized twice with WT BoHV-4 vector. Responses were assessed following peptide stimulation using an IFN-γ ELISpot assay. PBMC responses were assessed longitudinally **(A)**. On D63, PBMC responses to stimulation with peptide pools representing M/NSP5 from PRRSV-1 and -2 are compared **(B)**. Responses were additionally assessed in cells isolated from the BAL **(C)**, thymus **(D)**, inguinal lymph nodes **(E)**, and spleen **(F)**. Data are presented as the unstimulated condition-corrected number of IFN-γ spot-forming cells (S-C) per million cells **(B-F)**. Mean data ± SD are shown for **(A)**, whereas for **(B–F)**, data points represent individual pigs, with the median indicated by a horizontal line. *P < 0.05; **P < 0.005; ***P < 0.0005.

IFN-γ ELISpot assays were performed with freshly isolated PBMC collected weekly from D0 to D70 in Study 1. PRRSV-1 M/NSP5-specific responses were detectable after BoHV-4-M-NSP5 immunization, which were boosted by a second immunization (**Figure 1A**). The kinetics of the peptide-specific response was closely mirrored by spontaneous IFN-γ release from unstimulated cells, albeit at lower levels (**Figure 1B**). Statistical analyses revealed that for both BoHV-4-M-NSP5 prime and BoHV-4-M-NSP5 prime-boost groups, the number of cells secreting IFN-γ in response to peptide stimulation was significantly (*P <*0.05) greater than that without stimulation at all time points except D0 (*P >*0.05) and D56 (*P* = 0.08) for the BoHV-4-M-NSP5 prime-boost group, and D0 (*P >*0.05) for the BoHV-4-M-NSP5 prime group). In the WT BoHV-4 group, the number of IFN-γ-secreting cells with peptide stimulation was significantly (*P <*0.05) greater than that without stimulation only at D28 and then after the challenge (D49–D70) (**Figure 1A**). When evaluating unstimulated condition-corrected data over time, there was no significant NSP5/M-specific response in the WT BoHV-4 group (*P >*0.05), and the M/NSP5 response in the BoHV-4-M-NSP5 prime group was only significant on D70 compared to D0 (*P <*0.05). In contrast, the BoHV-4-M-NSP5 prime-boost group had a significantly greater IFN-*γ* response on D28 (7 days post-boost) than the other time points (*P <*0.05). Responses on D42 and D70 in the prime boost group were also higher than those on D0 (*P <*0.05). Intergroup comparisons revealed greater responses between the BoHV-4-M-NSP5 prime-boost group and the other two groups on D28, D35, and D42 (*P <*0.05). On D70, the responses in the two BoHV-4-M-NSP5 groups were higher than those in the BoHV-4 control group (*P <*0.05). On D63 (21 dpc), T-cell cross-reactivity was assessed by stimulating PBMC with M/NSP5 peptides representing PRRSV-1 and PRRSV-2 (**Figure 1B** and **Supplementary Figure 4A**). The number of IFN-*γ*-secreting cells following stimulation with either peptide pool was significantly higher than that without stimulation in all treatment groups (*P <*0.03), except for PRRSV-1 in the BoHV-4-M-NSP5 prime-only group (*P* = 0.06). The number of IFN*-γ*-secreting cells following stimulation with PRRSV-2 peptides was significantly higher than with PRRSV-1 peptides in the BoHV-4-M-NSP5 prime-boost group, but not in the other two groups, where the numbers did not significantly differ (*P* >0.05). For the compartmentalized tissue response (**Supplementary Figures 4B–E**), the number of IFN*-γ*-secreting cells following stimulation with M/NSP5 specific peptides was significantly higher than that without stimulation at 7 dpc (D49) and 28 dpc (D70) in all three treatment groups (*P <*0.05), except in the thymus of the WT BoHV-4 group at 7 dpc (*P* = 0.13). After media correction, no significant differences were observed between the groups in response to BALC (**Figure 1C**), thymus (**Figure 1D**), and inguinal lymph node (**Figure 1E**) cells. Spleen cells from pigs immunized with BoHV-4-M-NSP5 (prime-boost) showed greater responses than those from the BoHV-4 group (*P <*0.05) (**Figure 1F**).

### Assessment of efficacy of BoHV-4 vectored PRRSV-1 M and NSP5

The ability of BoHV-4-M-NSP5 to protect pigs against the PRRSV-1 subtype 1 isolate 21301/19 was assessed in Study 1. Pigs were euthanized at 7 or 28 dpc to assess the lung pathology.

#### Clinical signs

Most pigs had elevated rectal temperatures for approximately 3–6 days following PRRSV-1 21301/19 challenge (**Supplementary Figure 5A**). The number of pigs with elevated rectal temperature did not differ among the treatment groups for any of the time periods (*P >*0.16). Similarly, the duration of elevated rectal temperature did not differ among the treatment groups for any of the time periods (*P >*0.44). No other clinical signs were observed apart from a single pig in the BoHV-4-M-NSP5 prime group, which showed changes in social behavior and dyspnea (**Supplementary Figure 5B**).

#### Viral loads

**Figure 2:**
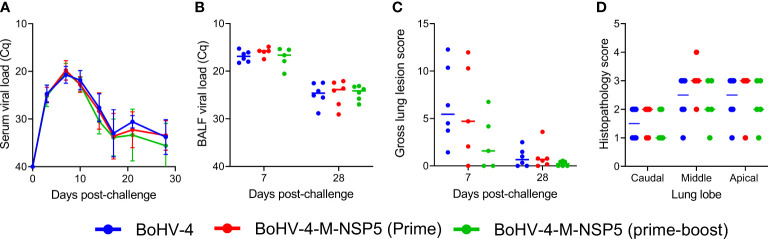
Evaluation of efficacy of BoHV-4-M-NSP5. The ability of BoHV-4-M-NSP5 immunization to confer protection was assessed following challenge infection of pigs with PRRSV-1 21301/19 (Study 1). The negative control group comprised two immunizations with BoHV-4 vector. PRRSV loads in the blood **(A)** and lungs **(B)** were inferred by RT-qPCR analysis of serum and BALF samples, respectively, and gross lung lesions **(C)** and lung histopathology (**D**; scores for 7 days post-challenge) were scored. Mean data ± SD are shown for **(A)**, whereas for **(B–D)**, data points represent individual pigs, with the median indicated by a horizontal line.

The levels of viremia in pigs following PRRSV-1 challenge were inferred using RT-qPCR. The level of viremia increased from 0 to 10 dpc, after which it declined, reaching a constant level from 17 dpc onward (**Figure 2A**). This temporal pattern in Cq values was the same across all treatment groups, i.e., there was no significant (*P* = 0.80) interaction between the treatment group and dpc. Moreover, the level of viremia at each time point did not differ significantly (*P* = 0.44) among the treatment groups. The viral load in BALF was measured using RT-qPCR in samples collected from the left lung at 7 and 28 dpc. Median C_q_ values were significantly higher at 7 dpc than at 28 dpc (*P <*0.004) but did not differ among treatment groups at either time point (*P >*0.42) (**Figure 2B**).

#### Lung pathology

The lungs collected at 7 and 28 dpc (D49 and D70) were digitally scored for gross lesions. There was a trend for higher lung lesion scores at 7 dpc than at 28 dpc and lower scores for the BoHV-4-M-NSP5 prime-boost group compared with the BoHV-4-M-NSP5 prime and WT BoHV-4 groups (**Figure 2C**). However, the median scores did not differ significantly among the treatment groups at the corresponding time points (*P >*0.21). Median scores differed significantly between 7 and 28 dpc only in the BoHV-4 group (*P* = 0.01). Microscopic lung lesions were also examined in H&E-stained sections of cranial, medial, and caudal lung lobes. There were no significant differences in the histopathological scores among the treatment groups (**Figure 2D**).

### Phenotyping of circulating effector cytokine-expressing PRRSV-1 M and NSP5 specific T cells

**Figure 3:**
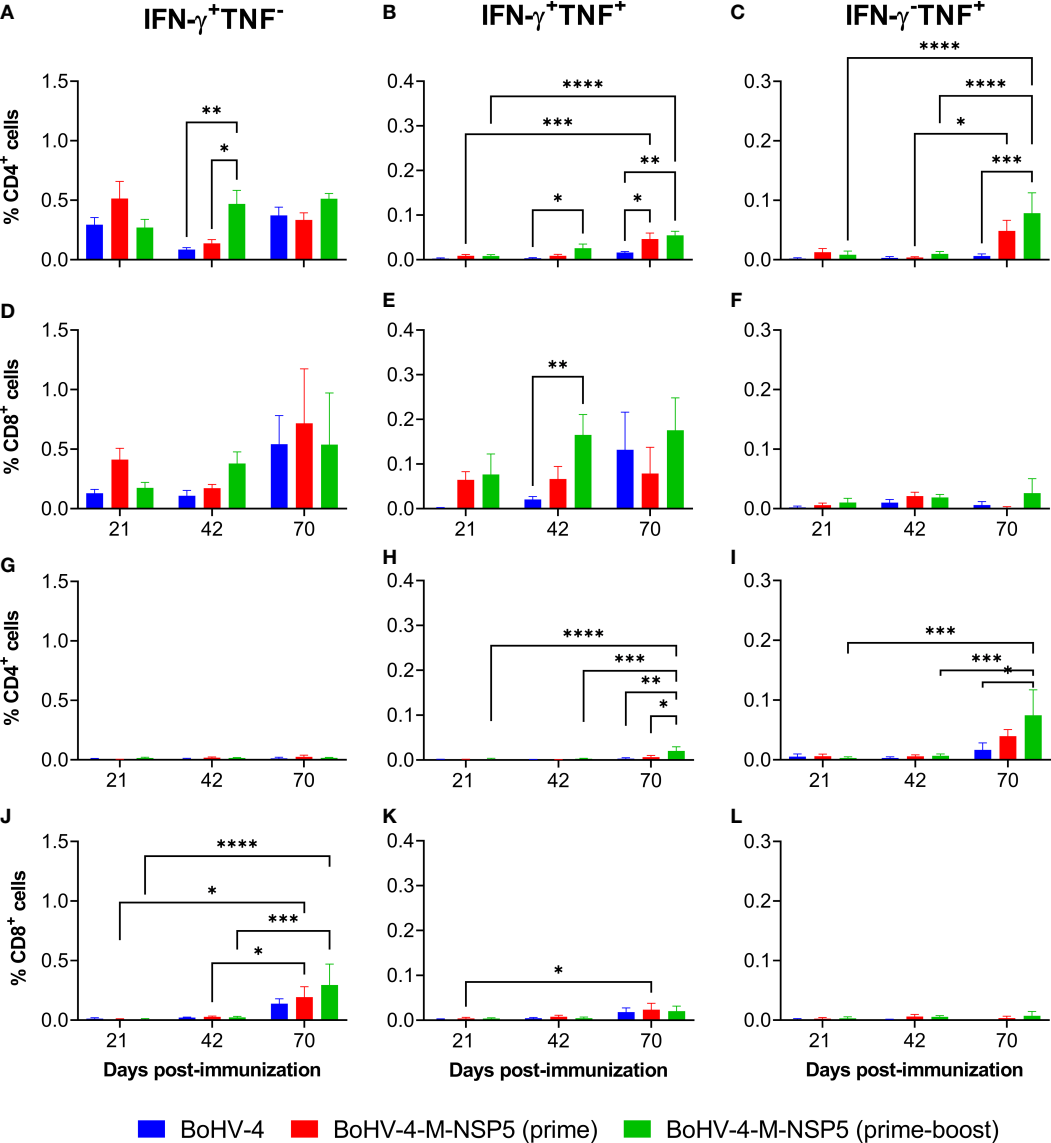
Characterization of antigen-specific cytokine responses in the blood of pigs after vaccination with BoHV-4-M-NSP5 and PRRSV-1. PRRSV-1 M/NSP5 specific cytokine responses were assessed following one (prime) or two (prime-boost) immunizations of pigs with BoHV-4-M-NSP5 and PRRSV-1 21301/19 (Study 1). Control pigs were immunized twice with the BoHV-4 vector before challenge. The responses of previously cryopreserved PBMCs from D21, D42, and D70 were assessed following peptide **(A–F)** and live PRRSV-1 21301/19 **(G–L)** stimulation using flow cytometry. Expression of IFN-γ alone **(A, D, G, J)**, IFN-γ and TNF **(B, E, H, K)**, or TNF alone **(C, F, I, L)** by CD4 T cells **(A–C, G–I)** and CD8 T cells **(D–F, J–L)** are shown as mean unstimulated condition-corrected data ± SD for each vaccine group. *P < 0.05; **P < 0.005; ***P < 0.0005; ****P < 0.0001.

To further examine the limited protective effect of BoHV-4-M-NSP5 immunization, the cellular sources of the cytokine responses were assessed. Previously cryopreserved PBMC from Study 1 were stimulated with PRRSV-1 M/NSP5 peptides and analyzed by flow cytometry (**Figures 3A–F**; cytokine expression with and without peptide stimulation is shown in **Supplementary Figures 6A–F**). Time points were selected to assess responses after priming (D21), boost (D42), and challenge (D70). IFN-γ- and TNF-expressing CD4^+^ or CD8^+^ T cells were classified as either single or dual (polyfunctional) cytokine-expressing cells (**Supplementary Figure 7**). In the BoHV-4-M-NSP5 prime-boost group, there was a significant increase in the frequency of IFN-γ^+^ TNF^+^ CD4^+^ T cells over time (D70 > D42 > D21; *P <*0.05), whereas no differences were noted in the WT BoHV-4 group (*P >*0.05). For the BoHV-4-M-NSP5 prime group, significant increases were found between D70, D21, and D42 (*P <*0.05; **Figure 3B**). Comparing the groups on D42, a significant increase in IFN-γ^+^ TNF^−^ (**Figure 3A**) and IFN-γ^+^ TNF^+^ (**Figure 3B**) CD4^+^ T cells was observed in BoHV-4-M-NSP5 prime-boost group compared to the other groups (*P <*0.05). After challenge (D70), the frequencies of IFN-γ^−^ TNF^+^ (**Figure 3C**) and IFN-γ^+^ TNF^+^ CD4^+^ T cells (**Figure 3B**) were greater in both BoHV-4-M-NSP5 vaccinated groups than in the control group (*P <*0.05). Analyses of CD8^+^ T cells showed a trend towards increased single IFN-γ and dual cytokine expression in the BoHV-4-M-NSP5 prime-boost group over time (*P >*0.05; **Figures 3D, E**). On D42, a greater frequency of IFN-γ^+^ TNF^+^ CD8^+^ T cells was observed in the BoHV-4-M-NSP5 prime-boost group than in the BoHV-4 group (*P <*0.05) (**Figure 3E**). No differences were observed in CD8^+^ T cells that produced only TNF (**Figure 3F**). To further investigate the polyfunctionality of the responding CD8^+^ T cells, perforin expression in single- and dual-cytokine-producing cells was assessed (**Supplementary Figure 8**). At D42 for pigs immunized twice with BoHV-4-M-NSP5, approximately half of the IFN-γ^+^ TNF^+^ CD8^+^ T cells co-expressed perforin, whereas most single cytokine-producing cells did not.

To assess the recognition of peptides presented by antigen-presenting cells processing challenge virus, previously cryopreserved Study 1 PBMC from D21, D42 and D70 were restimulated with PRRSV-1 21301-19 (**Figures 3G–L**; cytokine expression with and without PRRSV-1 stimulation is shown in **Supplementary Figure 9**). Significant CD4^+^ T-cell cytokine responses were only detected on D70, when the frequency of IFN-γ^+^ TNF^+^ cells in the BoHV-4-M-NSP5 prime-boost group, albeit low, was greater than that in the other groups (*P <*0.05) (**Figure 3H**), and IFN-γ^−^ TNF^+^ cells were greater than those in the control group (*P <*0.05) (**Figure 3I**). CD8^+^ T-cell IFN-γ responses to viral stimulation were also elevated on D70. While there were no significant differences between groups at this time point, only the BoHV-4-M-NSP5 immunized groups had a significantly greater frequency of IFN-γ^+^ TNF^−^ cells compared to the earlier time points (*P <*0.05) (**Figure 3J**). Compared to peptide stimulation, the overall weaker cytokine responses to PRRSV-1 stimulation, likely reflect the poor susceptibility of monocytes to infection, which limits the *de novo* expression and presentation of antigens (51, 75–77).

### Assessment of proliferative and IL-10 responses of PRRSV-1 M and NSP5 specific T cells

**Figure 4:**
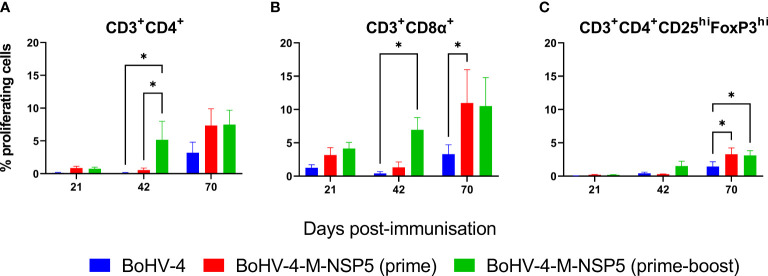
Characterization of antigen-specific proliferative responses in the blood of pigs after vaccination with BoHV-4-M-NSP5 and PRRSV-1. PRRSV-1 M/NSP5 specific proliferative responses were assessed following one (prime) or two (prime-boost) immunizations of pigs with BoHV-4-M-NSP5 and challenge with PRRSV-1 21301/19 (Study 1). Control pigs were immunized twice with the BoHV-4 vector before challenge. The responses of previously cryopreserved PBMCs from D21, D42, and D70 were assessed by flow cytometry following peptide stimulation. The mean unstimulated condition corrected % proliferation of CD4 T cells **(A)**, CD8α^+^ T cells **(B)**, and Tregs **(C)** is shown as ± SD for each vaccine group. *P < 0.05.

To further assess the effect of vaccination on T-cell activation and differentiation, the proliferative responses of PBMC from Study 1 were investigated after stimulation with M/NSP5 peptides (proliferative responses in the presence and absence of peptide stimulation are shown in **Supplementary Figure 10**). Flow cytometric analyses were used to identify proliferating CD4^+^ T cells, CD4^+^ regulatory T cells (Tregs), and CD8α^+^ T cells (which include both conventional CD8^+^ T cells and a subpopulation of γδ T cells (78)) (**Supplementary Figure 11**). As shown in **Figure 4A**, BoHV-4-M-NSP5 boost and PRRSV-1 challenge increased the proliferation of CD4^+^ T cells, with significant increases observed on D42 and D70 compared to D21 in the BoHV-4-M-NSP5 prime-boost group (*P <*0.05) (**Figure 4A**). On D42, greater CD4^+^ T-cell proliferation was observed in the BoHV-4-M-NSP5 prime-boost group than in the other groups (*P <*0.05). Higher frequencies of proliferating CD8α^+^ T cells were observed after challenge compared to D21 and D42 in BoHV-4-M-NSP5 prime-boosted animals (*P <*0.05) (**Figure 4B**). On both D42 and D70, greater CD8α^+^ T-cell proliferation was observed in both BoHV-4-M-NSP5 immunized groups compared than in the control BoHV-4 group (*P <*0.05). Treg cells (**Figure 4C**) followed a similar pattern to the total CD4^+^ T-cell population, with the frequency of proliferating cells increasing over time (D21 < D42 < D70) in pigs that received the BoHV-4-M-NSP5 prime-boost (*P <*0.05). An increased frequency of proliferating Treg cells was also seen on D70 when compared to D21 in the BoHV-4-M-NSP5 prime and control groups (*P <*0.05). At D70, there was a greater frequency of Treg cell proliferation in the vaccine group than in the control group (*P <*0.05). After peptide stimulation, there was also a trend for higher IL-10 production in the BoHV-4-M-NSP5 vaccination group, albeit without statistical significance (**Supplementary Figure 12**).

### Assessment of lung infiltrating T-cell responses

Since the lungs are heavily affected during PRRSV infection, the M/NSP5-specific T-cell response within the BALC was further characterized. First, phenotypic analysis was performed on T cells within BALC samples isolated at 7 (D49) and 28 (D70) dpc (**Supplementary Figure 13**). At both time points, there were no significant differences in the frequency of total T cells, CD4^+^ T cells, or CD8^+^ T cells among the three groups (**Supplementary Figures 14A–C**). At D49, non-conventional CD3^+^CD4^−^CD8α^−/low^ T cells (“γδ T cells”) were significantly higher in the BoHV-4-M-NSP5 prime-boost group than in the BoHV-4 control group (*P* = 0.04) (**Supplementary Figure 14D**). Evaluation of CD25 expression, upregulated on the surface of activated T cells, revealed that at 7 dpc, the proportion of CD25 expressing T cells (**Supplementary Figure 14E**), CD8^+^ T cells (**Supplementary Figure 14G**), and γδ T cells (**Supplementary Figure 14H**) from the BoHV-4-M-NSP5 prime-boost pigs was significantly higher than that in the WT BoHV-4 control group (*P* = 0.007, 0.009, and 0.05, respectively). In contrast, CD4^+^ T cells expressed high levels of CD25 in all groups at both time points, with no statistically significant differences between the groups (**Supplementary Figure 14F**). Conversely, Treg cells were significantly higher at D49 for both the BoHV-4-M-NSP5 prime and BoHV-4 groups than in the BoHV-4-M-NSP5 prime-boost group (*P* = 0.04 and 0.02, respectively; **Supplementary Figure 14I**).

**Figure 5:**
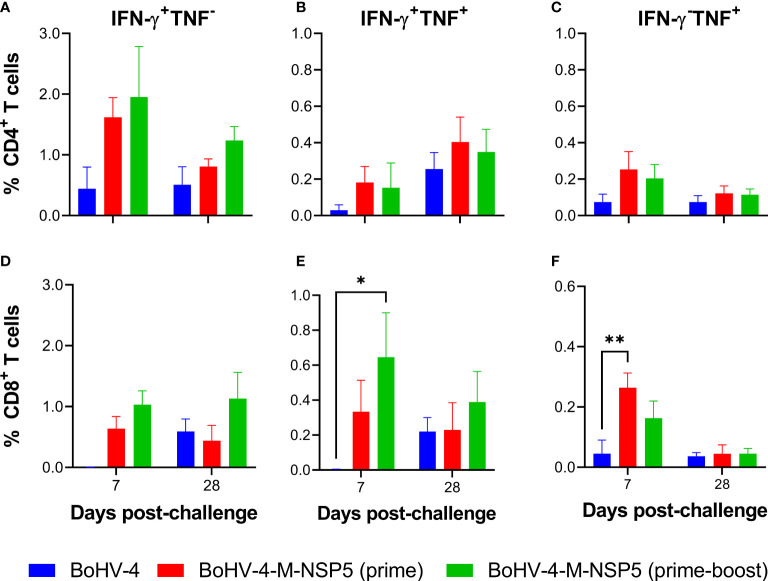
Characterization of antigen-specific IFN-γ and TNF responses in the lungs of pigs after vaccination with BoHV-4-M-NSP5 and PRRSV-1. PRRSV-1 M/NSP5 specific cytokine responses were assessed following one (prime) or two (prime-boost) immunizations of pigs with BoHV-4-M-NSP5 and PRRSV-1 21301/19 (Study 1). Control pigs were immunized twice with the BoHV-4 vector before the challenge. The responses of previously cryopreserved BALC from 7- and 28-days post-challenge were assessed following peptide stimulation by flow cytometry. Expression of IFN-γ alone **(A, D)**, IFN-γ and TNF **(B, E)**, or TNF alone **(C, F)**, by CD4 T cells **(A–C)** and CD8 T cells **(D–F)** are shown as mean unstimulated condition-corrected data ± SD for each vaccine group. *P < 0.05; **P < 0.005.

Finally, flow cytometry was employed to phenotype M/NSP5 specific BALC (**Supplementary Figure 15** and **Figure 5**). At 7 dpc, there was a significantly higher frequency of IFN-γ^+^ TNF^−^ CD4^+^ T cells (**Figure 5A**) in the BALC of pigs immunized with BoHV-4-M-NSP5 (prime-boost) than in those immunized with BoHV-4 (*P <*0.05). There was also a trend towards a higher frequency of polyfunctional and IFN-γ^−^ TNF^+^ CD4^+^ T cells (**Figures 5B, C**) in BALC from both BoHV-4-M-NSP5 immunized groups in the control group (*P >*0.05). Higher frequencies of IFN-γ^+^TNF^−^ (**Figure 5D**), although not statiscally significant, and IFN-γ^+^ TNF^+^ (*P* < 0.05) CD8^+^ T cells were observed in the prime-boost group than in the BoHV-4 group (**Figure 5E**). In addition, a single dose of BoHV-4-M-NSP5 promoted a greater frequency of IFN-γ^+^ TNF^+^ CD8^+^ T cells than BoHV-4 did (*P <*0.05) (**Figure 5F**). At 28 dpc, no significant differences were observed except for a trend for higher CD8^+^ T cells producing IFN-γ alone in BoHV-4-M-NSP5 prime-boosted animals (**Figure 5D**). In contrast to PBMC, CD8^+^ T cells responding to BALC were predominantly perforin-negative (**Supplementary Figure 16**).

### Assessment of antibody responses

Antigens prepared from PRRSV-1 infected cells were used in ELISA to assess antibodies in both the serum (**Supplementary Figure 17A**) and BALF (**Supplementary Figure 17B**). Antibody responses were undetectable in sera from all pre-challenge groups. However, on D70 (28 dpc) antibody reactivity to the crude PRRSV-1 antigen was greater in the animals immunized twice with BoHV-4-M-NSP5 expressing M/NSP5 than in those immunized with the wild-type BoHV-4 vector (*P <*0.05). Antibody reactivity was detected in D70 BALF samples, which did not differ significantly between the groups. PRRSV N protein-specific antibodies were analyzed in serum (**Supplementary Figure 17C**) and BALF (**Supplementary Figure 17D**). All animals showed antibody responses measurable in both serum and BALF, with no significant differences between the groups. PRRSV-neutralizing antibodies were assessed in the serum post-vaccination and post-challenge. No neutralization of either PRRSV-1 Olot/91 or 21301-19 was observed in any of the sera (data not shown).

### Assessment of efficacy of BoHV-4 vectored PRRSV-1 M and NSP5 after challenge with divergent PRRSV-1 strain LT-3

Since BoHV-4-M-NSP5 immunization induced T-cell responses associated with reduced lung pathology following PRRSV-1 challenge, we decided to perform a second efficacy study using a genetically divergent PRRSV-1 subtype 3 strain (LT-3). In this study (Study 2), pigs were immunized twice with BoHV-4-M-NSP5, a positive control group was immunized with a licensed PRRSV-1 MLV, and a negative control group was inoculated with DMEM, prior to challenge with PRRSV-1 LT-3. Pigs were euthanized 10–11 dpc to enable the postmortem examination of lung pathology. Protection against PRRSV-1 infection was assessed by measuring clinical signs, viral loads in the blood and lungs, and lung pathology.

#### Clinical signs

Following PRRSV-1 challenge, the mean body temperature of pigs in the BoHV-4-M-NSP5 prime-boost group (39.5 °C) was significantly higher (*P* = 0.05) than unvaccinated (39.2 °C) and MLV group (39.2 °C), but this was not considered clinically relevant (**Supplementary Figure 18A**). Changes in social behavior and dyspnea scores were observed, and a stratified analysis revealed a trend of higher dyspnea scores in the BoHV-4-M-NSP5 group than in the MLV and unvaccinated groups (*P* = 0.08) and higher altered social behavior scores in the unvaccinated and BoHV-4-M-NSP5 groups than in the MLV group (*P <*0.01) (**Supplementary Figure 18B**).

#### Viral loads

**Figure 6:**
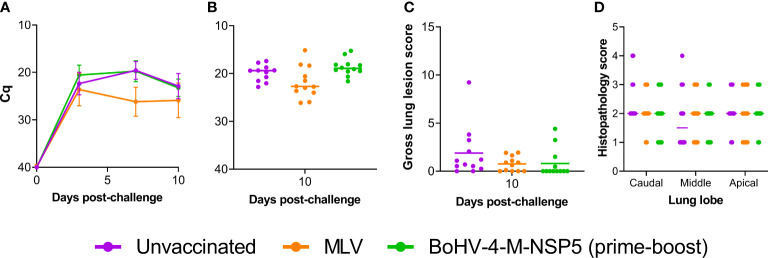
Evaluation of the efficacy of the BoHV-4 vectored PRRSV vaccine candidate. The ability of BoHV-4-M-NSP5 immunization to confer protection was assessed following challenge infection of pigs with the divergent strain LT-3 (Study 2). PRRSV loads in the blood **(A)** and lungs **(B)** were inferred from RT-qPCR analysis of serum and BALF samples, respectively, and gross lung lesions **(C)** and lung histopathology (**D;** Study 2 scores for 10 days post-challenge) were scored. Mean data ± SD are shown for **(A)**, whereas for **(B–D)**, data points represent individual pigs, with the mean indicated by a horizontal line.

Comparisons of C_q_ values measured on 0, 3, 7, and 10 dpc showed that there was no statistical significance between the unvaccinated and BoHV-4-M-NSP5 groups (*P >*0.05), whereas the MLV group had statistically lower viremia than the unvaccinated group at 3 dpc (P = 0.01) and lower than both unvaccinated and BoHV-4-M-NSP5 groups at 7 (P <0.001) and 10 dpc (P = 0.03) (**Figure 6A**). The viral load in BALF was measured by RT-qPCR in samples collected from the left lung lobe post-mortem. Median C_q_ values did not differ amongst treatment groups (*P >*0.05) (**Figure 6B**).

#### Lung pathology

There was no significant difference in gross lung lesions between the groups (*P >*0.10; **Figure 6C**), although there was a trend towards lower scores in the MLV and BoHV-4-M-NSP5 groups, with most animals in the BoHV-4-M-NSP5 group not presenting any lesions. There were no significant differences in the histopathological scores among the treatment groups (**Figure 6D**).

## Discussion

It has been hypothesized that cellular responses play an important role in immunity against PRRSV in the absence of neutralizing antibodies (34, 35, 79). The M and NSP5 proteins are conserved targets of polyfunctional T cells from PRRSV-1 immune pigs (51). CD8^+^ cells are the predominant T cell subset infiltrating the lungs of infected pigs, and CD4^+^ T helper cells in blood and lymphoid tissues coincide with reduced viremia (34–37). IFN-γ has been shown to reduce PRRSV infection in macrophages *in vitro* and IFN-γ responses have been associated with more effective clearance of PRRSV *in vivo* (39–42). Herpes viral vectors have been shown to enhance T-cell responses against heterologous target antigens (58–60). For example, a BoHV-4 vector expressing Nipah virus glycoproteins evoked potent antigen-specific CD4 and CD8 T-cell responses in pigs (62).Therefore, we aimed to exploit this property and test whether T-cell responses elicited by a BoHV-4 vector expressing PRRSV-1 M and NSP5 could protect pigs.

BoHV-4-M-NSP5 immunization did not induce any adverse effects. The low-level detection of nucleic acids in some swabs (including those from sentinel pigs) collected immediately prior to the booster inoculations (D21) was unexpected and may be an artfact due to environmental contamination. BoHV-4-M-NSP5 vaccination induced robust IFN-γ responses but did not induce an antibody response measurable pre-challenge. Indeed, the vaccine induced a response that was measurable by spontaneous IFN-γ release from isolated PBMC; however, a significant PRRSV-1 M-NSP5 specific response was also observed. This was most prominent after booster immunization. The negative control vector-only group displayed an M-NSP5 specific IFN-γ response post-challenge, albeit at a lower magnitude. The recall/boost of M-NSP5 specific IFN-γ responses was delayed and kinetically broadly mirrored the primary response in the control group. The induced T-cell responses were broadly reactive, with comparable responses observed in response to PBMC stimulation with PRRSV-2 M-NSP5 peptides. Post-challenge, high frequencies of peptide-specific IFN-γ-secreting cells were isolated from the lungs and spleens of BoHV-4-M-NSP5 vaccinated pigs, with lower frequencies observed in cells isolated from the inguinal lymph nodes and thymus. The effect of vaccination against challenge with two divergent pathogenic strains of PRRSV-1, measured by viremia and gross lung lesions, was not different between one or two doses of BoHV-4-M-NSP5 compared to the negative control groups. There were numerically, but not statistically significant, lower mean lung gross lesion scores in BoHV-4-M-NSP5 vaccinated animals than in the negative control groups. Further assessment of the phenotype and function of vaccine induced M-NSP5 specific T cells was conducted to elucidate factors that may have contributed to the protective limitations observed following PRRSV challenge.

Flow cytometric analyses showed that immunization with BoHV-4-M-NSP5 induced specific CD4^+^ and CD8^+^ T-cell responses in the blood, which were greater for IFN-γ^+^ TNF^+^ expression following prime-boost immunization. The M and NSP5 proteins have been shown previously to stimulate dual IFN-γ and TNF expressing cells following experimental PRRSV-1 infection (51), and it is thought that simultaneous ‘polyfunctional’ secretion of both cytokines provides increased robustness of the T-cell response (80, 81). In addition, it has been shown following PRRSV-1 infection that NSP5-specific CD8^+^ T cells and memory M-specific CD4^+^ T cells were generated (51). Further analysis of BoHV-4-M-NSP5 responding T cells could be undertaken to elucidate the relative contribution of antigenic regions stimulating T-cell subsets. IFN-γ^+^ TNF^+^-expressing CD4^+^ and CD8^+^ T cells were also shown to be present in the lungs following PRRSV-1 challenge in BoHV-4-M-NSP5 prime-boost-immunized pigs. Phenotyping of BALC also showed a high composition of CD8^+^ T cells relative to total live cells localized in the lungs for all vaccination groups, and these CD8^+^ T cells had increased CD25 expression in BoHV-4-M-NSP5 prime-boost pigs. Upregulation of CD25, the alpha-chain portion of the IL-2 receptor, has been associated with terminal effector differentiation, suggesting that CD8^+^ T cells in prime-boosted pigs are more activated (82, 83). The recruitment of such polyfunctional CD8^+^ T cells to the lungs and mucosa is thought to be crucial for PRRSV clearance from the lungs (81, 84). Indeed, a study using a PRRSV vaccine constructed with a hydrophobic chitosan-based particulate formulation suggested that the lack of CD8^+^ T-cell induction and effective cross-presentation was a key factor in the poor efficacy observed (50). The CD8^+^ T cells shown here to be increasingly present and functional in the lungs of prime-boost pigs could be one population responsible for the differences seen in lung lesion scores in this study, via infiltration to the tissue effector site. Assessment of perforin expression, as marker of cytotoxic potential, was incorporated along with cytokine expression analysis. While an increase in the proportion of perforin-expressing M-NSP5-specific CD8^+^ T cells was observed in cells circulating in the blood, perforin-expressing specific CD8^+^ T cells were not isolated from BAL. While it cannot be excluded that this technical artifact reflects the degranulation of more activated CD8^+^ T cells from BAL, it is tempting to speculate that perforin expression may be downregulated in PRRSV-infected lungs. Although technically demanding, determination of the cytotoxic activity of CD8^+^ T cells against PRRSV-infected macrophages should be undertaken to directly assess the functional capacity of these cells in the in blood and BAL (31). A previous study on CD8^+^ T cells from PRRSV-infected pigs showed that these cells had impaired cytotoxic activity (85). If BoHV-4-M-NSP5 primed CD8^+^ T cells were unable to kill PRRSV-infected macrophages, this could explain the limited effect of the vaccine on reducing PRRSV loads.

The failure of the PRRSV-1 challenge to rapidly recall vaccine-induced T-cell responses was another finding that may, in part, explain the inability of the response to control infection. The primary T-cell response to PRRSV infection is often characterized as ‘late’ in relation to infection kinetics (6), as reflected in the responses observed in the negative control group. It is unclear why PRRSV infection initially constrains the primary T-cell response and whether this could affect the recall of a memory response. However, during PRRSV-2 infection, the production of the immunosuppressive cytokine, interleukin-10 (IL-10) has been proposed to drive Treg development, which may consequently reduce effector T-cell responses (86), as has been reported for a number of human viruses (87–90). It has also been demonstrated in mice that higher IL-10 producing Treg cells reduce the efficacy of protective CD8^+^ T-cell responses (87, 91). An alternative hypothesis is that the robustness of the vaccine-induced T-cell response led to a degree of exhaustion or regulation that restricts the recall response post-challenge. In the absence of porcine T-cell exhaustion markers, we assessed the proliferative capacity of T cells, including Treg cells, post-prime, boost, and challenge. CD4 and CD8 T cells and Treg cells from BoHV-4-M-NSP5 prime-boost immunized pigs demonstrated significant proliferative capacity in response to antigens at the point of PRRSV challenge (D42). While this may suggest the ability of these cells to respond to challenge infection, we may only speculate as to whether the frequency of specific Tregs measurable in this assay could potentially restrain effector T-cell responses *in vivo*.

To further investigate the trend for reduced lung pathology in the absence of a concordant reduction in BALF viral loads, we phenotyped the T-cell populations infiltrating the lungs post-challenge. Treg cells were present at a significantly lower frequency in the lungs of BoHV-4-M-NSP5 prime-boosted pigs than in other groups. In contrast, a higher frequency of total and activated (CD25^+^) non-conventional T cells, predominantly γδ T cells (92), was present in the BALF from the BoHV-4-M-NSP5 prime-boost group. It has been shown previously that γδ T cells are one of the main immune response contributors post-PRRSV-viremia with high proliferation and partial IFN-γ production (34), although this was not observed in blood or lung γδ T cells (data not shown). It has been reported that γδ T cells play a role in lung homeostasis in various infectious diseases (93, 94), and it has been proposed that in PRRS they may have a proinflammatory role in innate immunity, including IFN-γ-producing γδ T cells in the lung, supporting macrophage activation (32, 34). However, a recent study using a neonatal mouse model of influenza demonstrated a role for γδ T cells in protection against disease, independent of virus control (95). It could therefore be speculated that γδ T cells, activated in part through vaccine-induced T-cell responses, may play a role in protecting the lungs of prime-boost immunized pigs against PRRSV-induced pathology through an undetermined mechanism.

In conclusion, our results here demonstrate that prime-boost immunization with the BoHV-4-M-NSP5 vaccine induced CD4 and CD8 T cell responses that were incapable of controlling PRRSV infection but were associated with a trend towards reduced lung pathology. These results suggest that a balanced T-cell and antibody response may be essential for protection, and future efforts to develop next-generation vaccines should focus on achieving this goal.

## Data availability statement

The original contributions presented in the study are included in the article/**Supplementary Material**. Further inquiries can be directed to the corresponding author.

## Ethics statement

The animal study was reviewed and approved by the Pirbright Institute, Pirbright, UK, Animal and Plant Health Agency, Addlestone, UK, and Poulpharm, Izegem, Belgium.

## Author contributions

HB, JS, VF, MJ, and SG acquired funding for the project. BS-B, JS, VF, MF, MH, HB, MJ, and SG contributed to the conception, design, and coordination of the study. RB, KH, JR, JS, YW, SH, TM, J-PF, MB, FL, AN, SG, NS, AD, MJ, and SG acquired, analyzed, and interpreted the data. RB, JS, and SG wrote the first draft of the manuscript. B-SB and MJ edited and revised the manuscript. All authors contributed to the article and approved the submitted version.
