# Supplementary material for: An attenuated herpesvirus vectored vaccine candidate induces T-cell responses against highly conserved porcine reproductive and respiratory syndrome virus M and NSP5 proteins that are unable to control infection
Source: Front Immunol. 2023 Aug 3;14:1201973. doi: 10.3389/fimmu.2023.1201973 (PMC10436000; doi:10.3389/fimmu.2023.1201973)
Supplement: Supplementary file 1 [file DataSheet_1.pdf]

Supplementary Material

An attenuated herpesvirus vectored vaccine candidate induces T cell responses against highly conserved porcine reproductive and respiratory syndrome virus M and NSP5 proteins that are unable to control infection

Rory C.F. de Brito, Kerry Holtham, Jessica Roser, Jack E. Saunders, Yvonne Wezel, Summer Henderson, Thekla Mauch, Beatriz Sanz-Bernardo, Jean-Pierre Frossard, Matthieu Bernard, Fabian Z. X. Lean, Alejandro Nunez, Simon Gubbins, Nicolás M. Suárez, Andrew J. Davison, Michael J. Francis, Michael Huether, Hafid Benchaoui, Jeremy Salt, Veronica L. Fowler, Michael A. Jarvis, Simon P. Graham

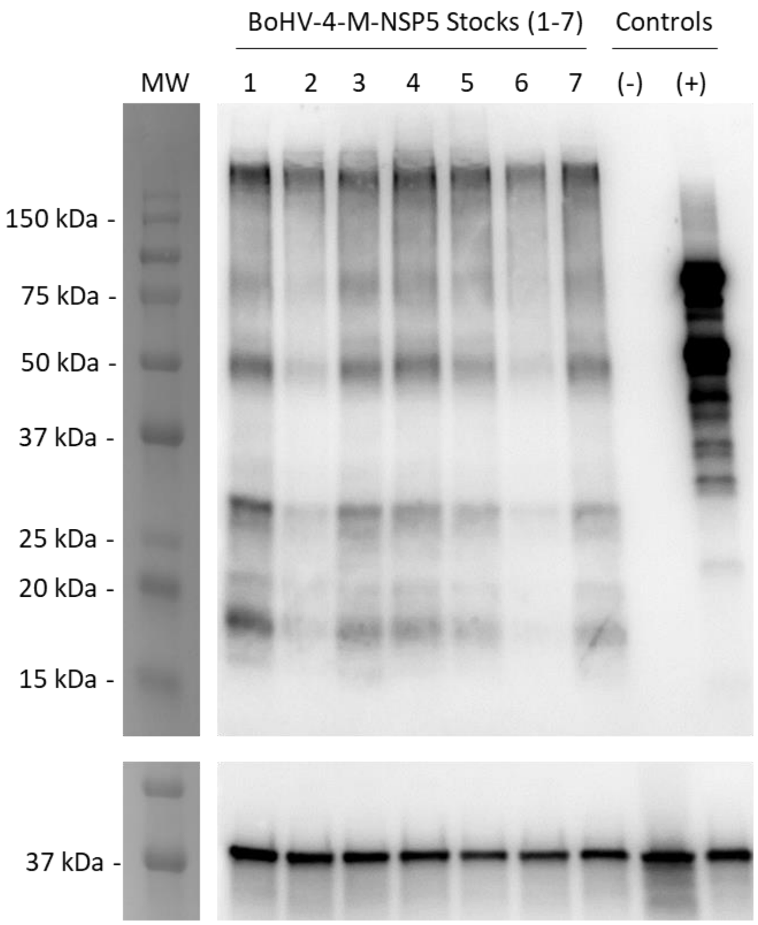

Supplementary Figure 1. Western immunoblot showing expression of M-NSP5 fusion protein by BoHV-4-M-NSP5. MDBK cells were infected with concentrated BoHV-4-M-NSP5 virus stocks (stocks 1-7). The figure shows western immunoblot analysis of cell lysates with multiple V5-reactive bands, in addition to the expected band size of 50 kDa, likely due to protein multimerization and proteolysis. Lysates were not heated to avoid excessive M-NSP5 aggregation. M-NSP5 was detected using an antibody directed against the C-terminal V5 epitope tag. A BoHV-4 WT (-) was used as a negative control and a BoHV-4 recombinant expressing a V5-tagged bovine tuberculosis antigen (+) was used as a positive control. An antibody directed against cellular p38 protein was used as a loading control.

Supplementary Table 1 – For study 1, the clinical score system comprised the below parameters measured daily for a week after each vaccination and for a maximum of 14 days post-challenge.

Parameter		*Criteria*		**Score**
**1**	**Skin changes**	1a. Redness/ purpling of skin of ears	Absent/Present	***0/1***
		1b.Reddening/purpling of skin elsewhere on body	Absent/Present	***0/1***
		1c. Ear necrosis	Absent/Present	***0/2***
**2**	**Behaviour**	2a. Alertness/Movement	Get up quickly look alert to what is happening/eyes open	***0***
			Lethargic	***1***
			Get up slowly not as alert, eyes closed	***2***
			No attempt to get up even when touched, eyes closed	***6***
		2b. Interaction with others	Normal	***0***
			Reduced	***1***
			None	***2***
		2c. Investigating toys	Keen interest in toys/rope	***0***
			Limited interest in toys/rope	***1***
			No interest	***2***
**3**	**Eyes/conjunctiva**	3a. Ocular discharge	Absent/Present	***0/1***
		3b. Swelling of eyelids	Absent/Present	***0/1***
**4**	**Nasal discharge**		Absent	***0***
			Present and clear	***1***
			Present and discoloured	***2***
**5**	**Respiratory changes**	Frequency 10-15/min, barely visible chest movement		***0***
		Frequency >20/min distinct chest and abdominal movement		***2***
		Frequency >30/min, breathing through open mouth		***6***
**6**	**Coughing**	None		***0***
		Mild (few incidences as 1-5)		***1***
		Severe (more frequent incidences >5)		***2***
**7**	**Defecation**	Soft faeces, normal amount		***0***
		Reduced amount of faeces, dry		***1***
		No faeces, mucus in rectum or diarrhoea		***2***
**8**	**Appetite**	Hungry, trough empty, clean		***0***
		Reduced eating		***1***
		Only picking at food		***4***
		Does not eat when fed		***6***
**9**	**Body Condition**	Good		***0***
		Fair		***1***
		Poor (ribs and/or backbone showing)		***2***
**10**	**Temperature**			
**11**	**Other**	Note any additional observations:		

Supplementary Table 2 – For study 2, the clinical score system comprised the below parameters measured daily for a week after each vaccination and for a maximum of 10 days post-challenge.

	Parameter	Criteria	Score
1	Skin changes		
1a	Reddening/purpling of skin of ears	Absent/present	0/1
1b	Reddening/purpling of skin elsewhere on body	Absent/present	0/1
1c	Ear necrosis	Absent/present	0/2
2	Behaviour		
2a	Alertness/Movement	Get up quickly look alert to what is happening/eyes open	0
		Lethargic	1
		Get up slowly not as alert, eyes closed	2
		No attempt to get up even when touched, eyes closed	6
2b	Interaction with others	Normal	0
		Reduced	1
		None	2
2c	Investigating toys	Keen interest in toys/rope	0
		Limited interest in toys/rope	1
		No interest	2
3	Eyes/conjunctiva		
3a	Ocular discharge	Absent/Present	0/1
3b	Swelling of eyelids	Absent/Present	0/1
4	Nasal discharge	Absent	0
		Present and clear	1
		Present and discoloured	2
5	Respiratory changes	Frequency 10-15/min, barely visible chest movement	0
		Frequency >20/min distinct chest and abdominal movement	2
		Frequency >30/min, breathing through open mouth	6
6	Coughing	None	0
		Mild (few incidences as 1-5)	1
		Severe (more frequent incidences >5)	2
7	Defecation	Soft faeces, normal amount	0
		Reduced amount of faeces, dry	1
		No faeces, mucus in rectum or diarrhoea	2
8	Appetite	Hungry, trough empty, clean	0
		Reduced eating	1
		Only picking at food	4
		Does not eat when fed	6
9	Body Condition	Good	0
		Fair	1
		Poor (ribs and/or backbone showing)	2
10	Other	Note any additional observations	

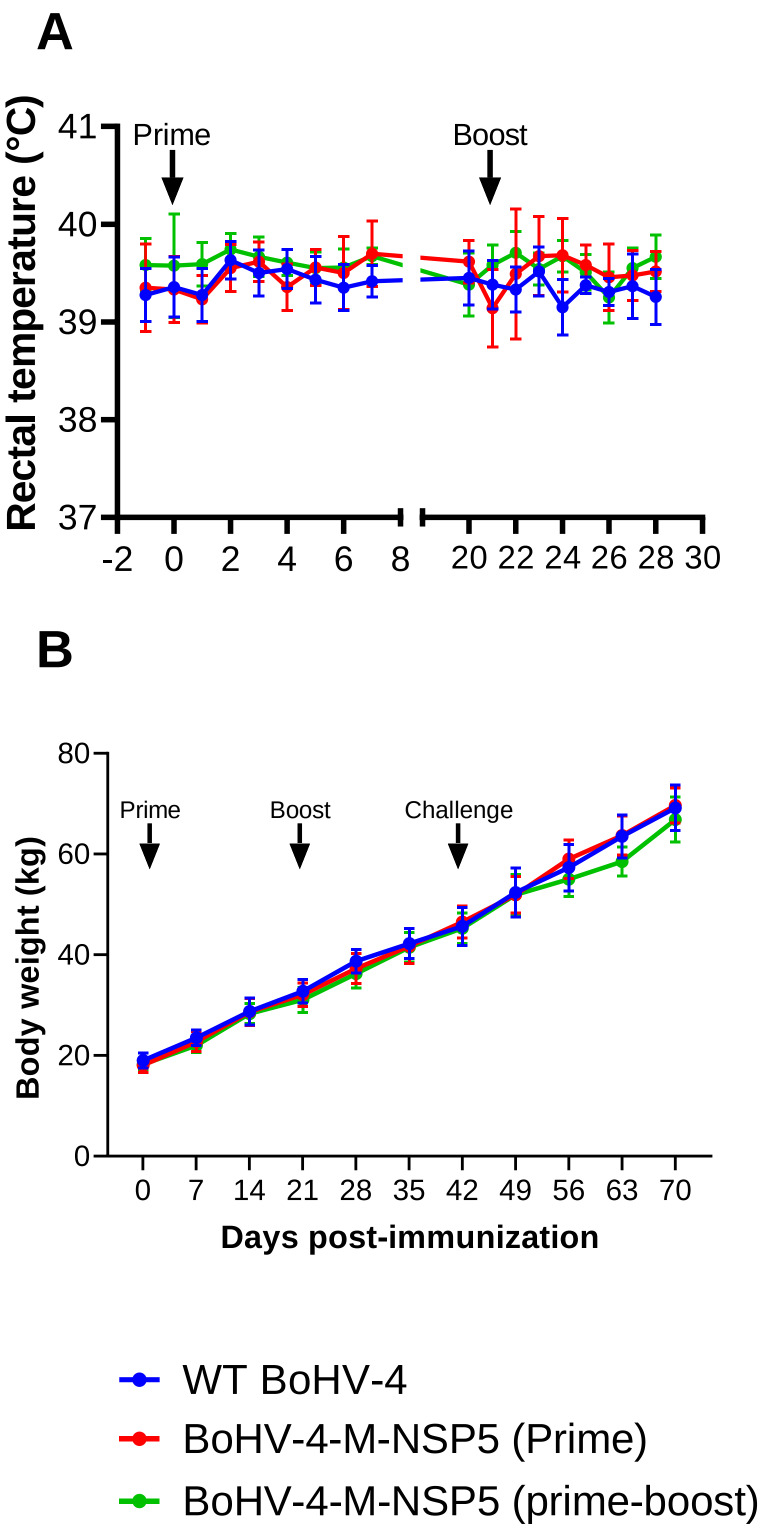

Supplementary Figure 2. Evaluation of the safety of a BoHV-4 vectored PRRSV vaccine candidate. In study 1, rectal temperatures measured daily for 7 days post-prime and -boost immunization (**A**). Animals were weighed weekly post-vaccination and -challenge **(B)**. Mean data ± SD for each group are shown.

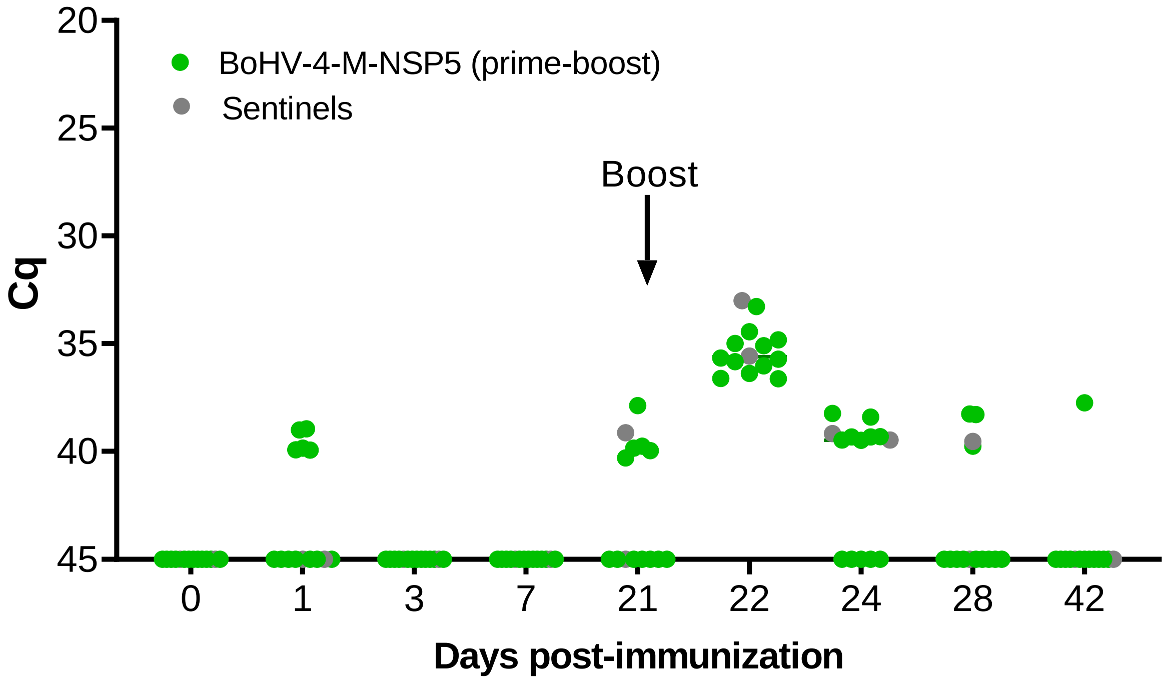

Supplementary Figure 3. Assessment of BoHV-4 shedding genome detection in nasal swabs. BoHV-4 genome in nasal swabs was inferred by qPCR analysis of pigs immunized with BoHV-4-M-NSP5 (prime-boost) from study 1. Swabs from co-housed sentinel pigs were included in the analysis. Data are presented as C_q values, datapoints represent individual pigs with the median indicated by a horizontal line.

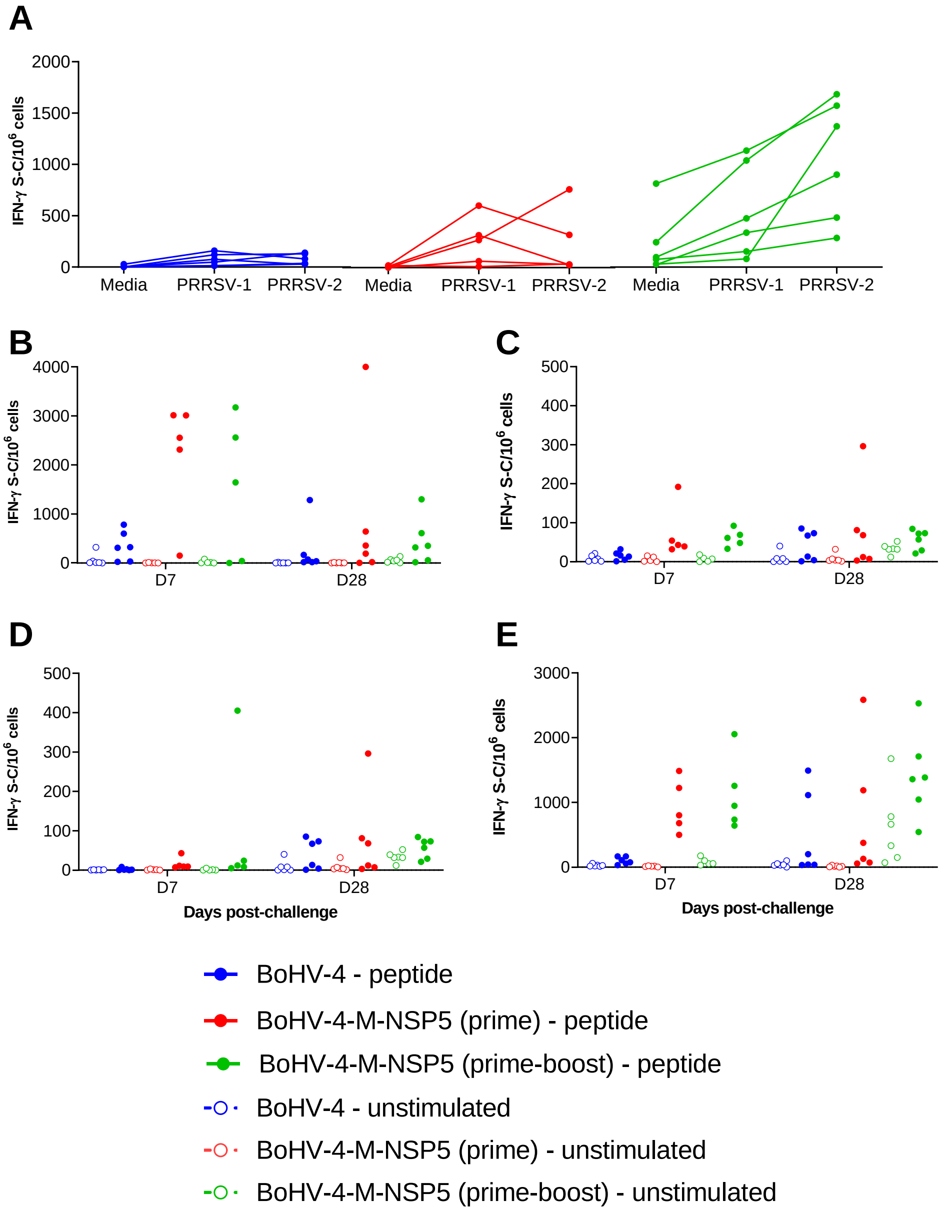

Supplementary Figure 4. Assessment of IFN-γ responses induced by immunization with a BoHV-4 vectored PRRSV vaccine candidate and PRRSV-1 challenge. PRRSV-1 M/NSP5 specific IFN-γ responses were assessed following one (prime) or two (prime-boost) immunizations of pigs with BoHV-4-M-NSP5 and challenge with PRRSV-1 21301/19 (study 1). Control pigs were immunized twice with the WT BoHV-4 vector. PBMC responses were assessed following peptide stimulation by IFN-γ ELISpot assay. PBMC responses were assessed on D63 by stimulation with peptide pools representing M/NSP5 from PRRSV-1 and -2 (**A**). Responses were additionally assessed from cells isolated from BAL (**B**), thymus (**C**), inguinal lymph nodes (**D**) and spleen (**E**) post-challenge. Datapoints represent the unstimulated or peptide-stimulated IFN-γ spot-forming cells (S-C) per million cells for individual pigs.

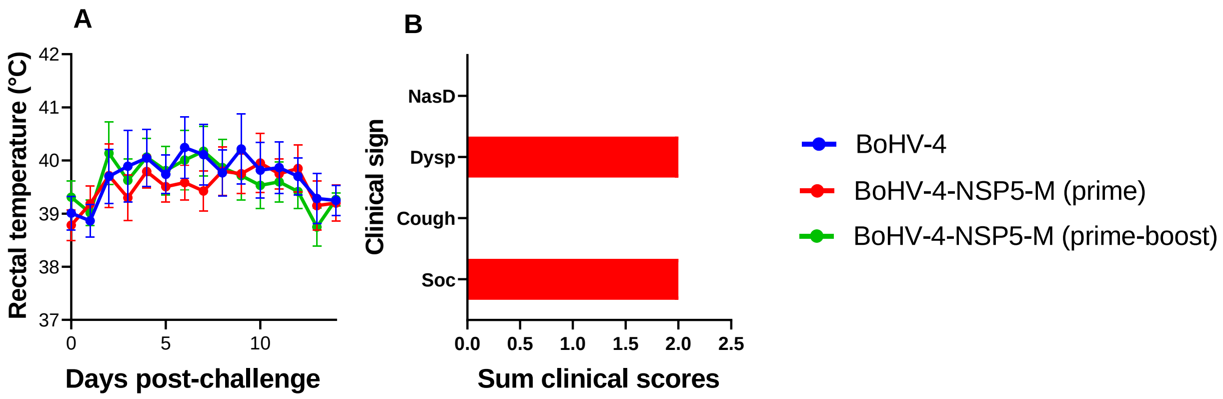

Supplementary Figure 5. Evaluation of the efficacy of a BoHV-4 vectored PRRSV vaccine candidate. The ability BoHV-4-M-NSP5 immunization to confer clinical protection was assessed following challenge infection of pigs with PRRSV-1 21301/19 (Study 1). A negative control group comprised two immunisations with the BoHV-4 vector. Mean data ± SD for rectal temperatures over the course of challenge are shown (**A**). The sum of clinical scores observed in the post-challenge period are presented for each group (**B**). Nasal discharge – NasD; dyspnoea – Dysp, and changes in social behavior – Soc.

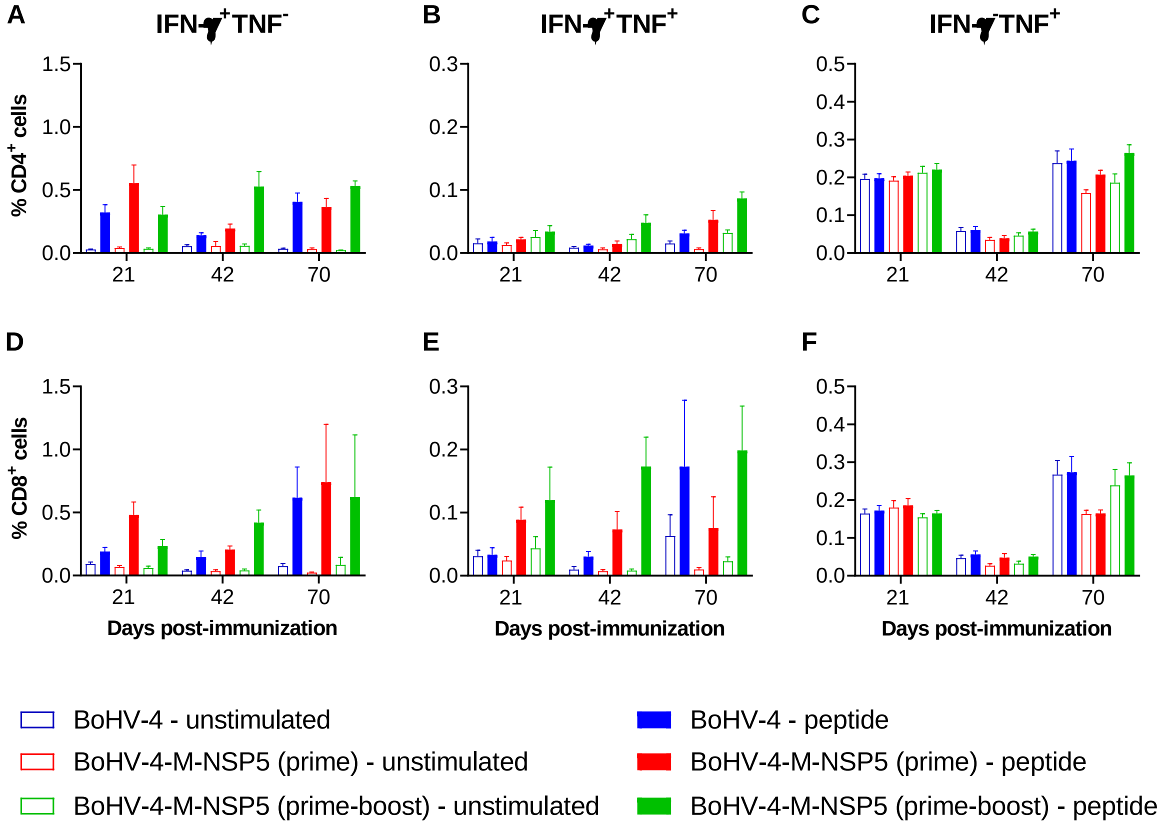

Supplementary Figure 6. Characterization of antigen-specific cytokine responses in the blood of pigs after vaccination with BoHV-4-M-NSP5 and PRRSV-1 challenge. PRRSV-1 M/NSP5 specific cytokine responses were assessed following one (prime) or two (prime-boost) immunizations of pigs with BoHV-4-M-NSP5 and challenge with PRRSV-1 21301/19 (Study 1). Control pigs were immunized twice with the BoHV-4 vector before the challenge. Responses of previously cryopreserved PBMCs from D21, D42 and D70 were assessed with and without peptide stimulation by flow cytometry. Expression of IFN-γ alone (**A**, **D**), IFN-γ and TNF (**B, E**), or TNF alone (**C**, **F**) by CD4 T cells (**A - C**) and CD8 T cells (**D - F**) are shown as mean data ± SD for each vaccine group.

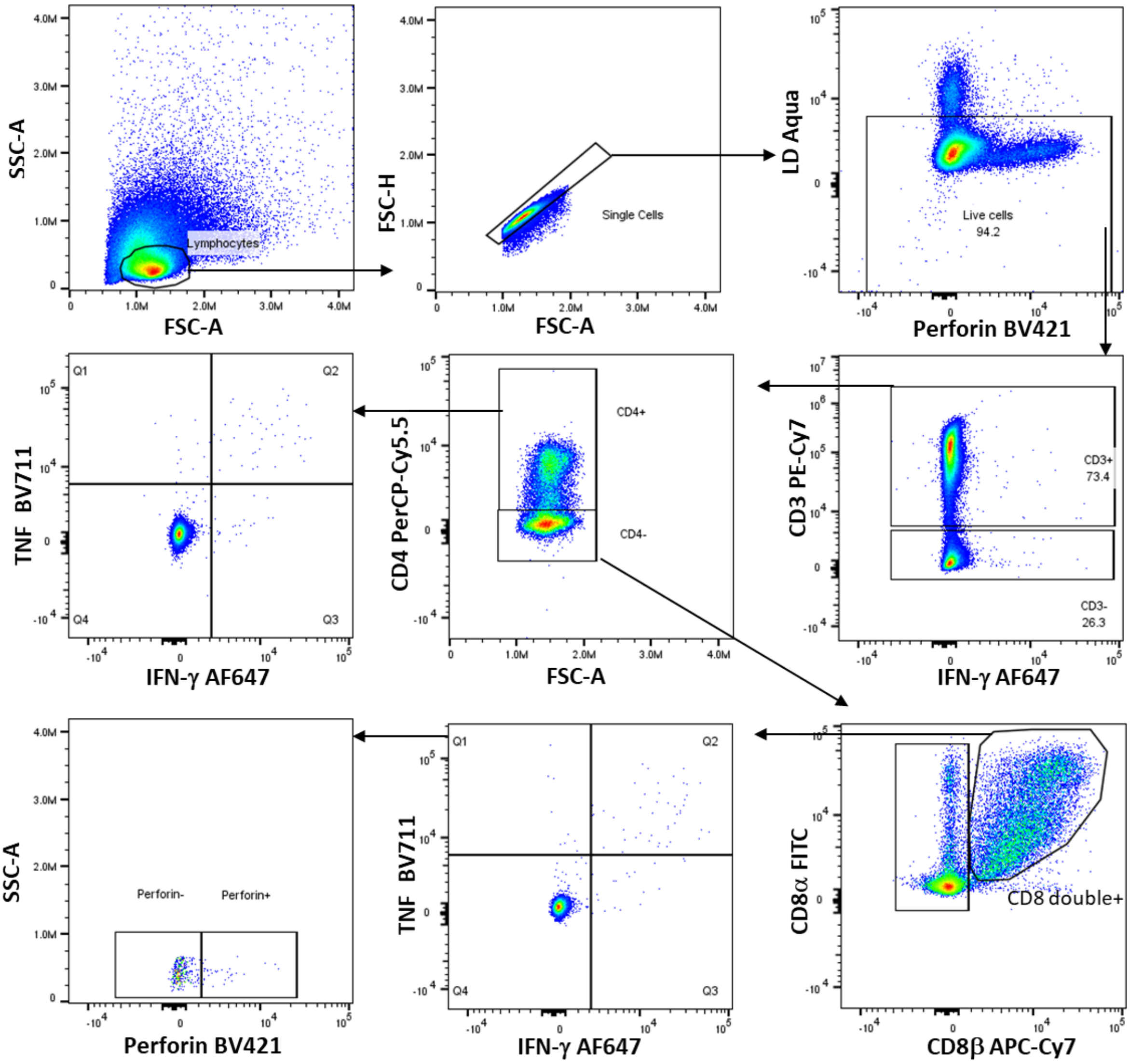

Supplementary Figure 7. Flow cytometric gating strategy for assessment of T cell cytokine responses. Lymphocytes were gated by their characteristic light scattering and then further gated to exclude dead cells. Live lymphocytes were separated into CD3^{+} T-cells and CD3^{-} non-T-cells before CD3^{+} cells were sub-gated for CD4^{+} T cells, CD8αβ^{+} T cells, non-conventional T cells defined as CD3^{+} CD8α$^{low/+}$ CD8β^{-} CD4^{-} T-cells. Single and double IFN-γ^{+} and TNF^{+} cells were gated within the various CD3^{+} populations. Finally, the perforin expression by cytokine expressing CD8 T cells was assessed.

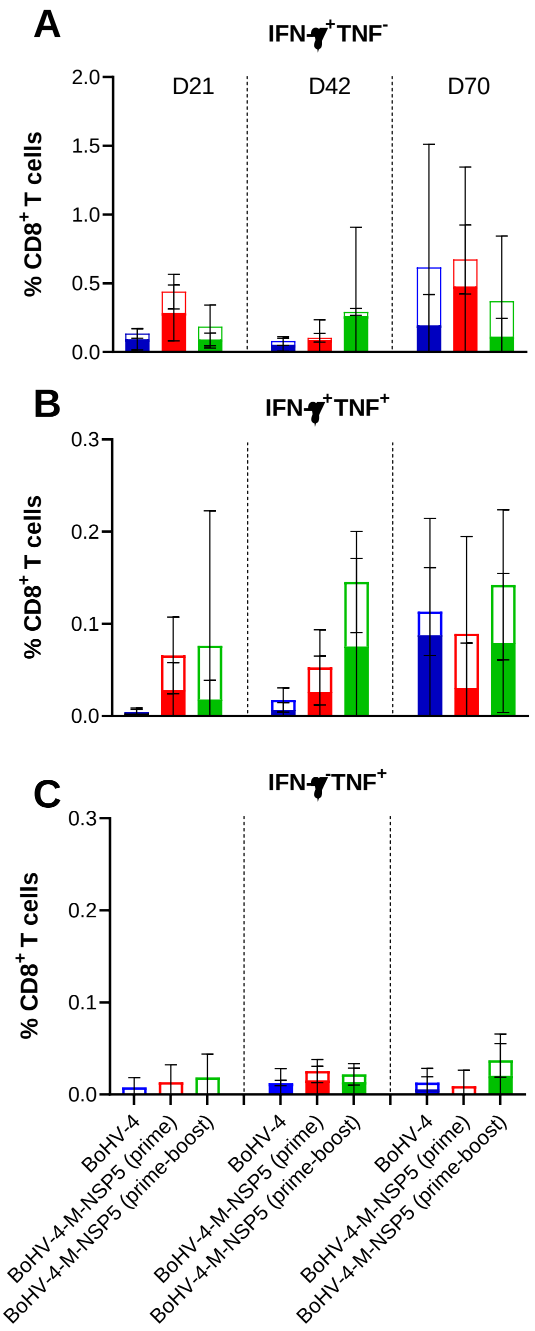

Supplementary Figure 8. Assessment of perforin expression by CD8 T cells after vaccination with BoHV-4-M-NSP5 and PRRSV-1 challenge. PRRSV-1 M/NSP5 specific cytokine responses were assessed following one (prime) or two (prime-boost) immunizations of pigs with BoHV-4-M-NSP5 and challenge with PRRSV-1 21301/19 (study 1). Control pigs were immunized twice with the BoHV-4 vector before PRRSV challenge. Responses of previously cryopreserved PBMCs from D21, D42 and D70 were assessed following peptide stimulation by flow cytometry. Perforin expression (perforin$^+$ - empty bars; perforin$^-$ - solid bars) by CD8 T cells expressing IFN-γ alone (**A**), IFN-γ and TNF (**B**), or TNF alone (**C**) are shown as mean unstimulated condition corrected data ± SEM for each vaccine group.

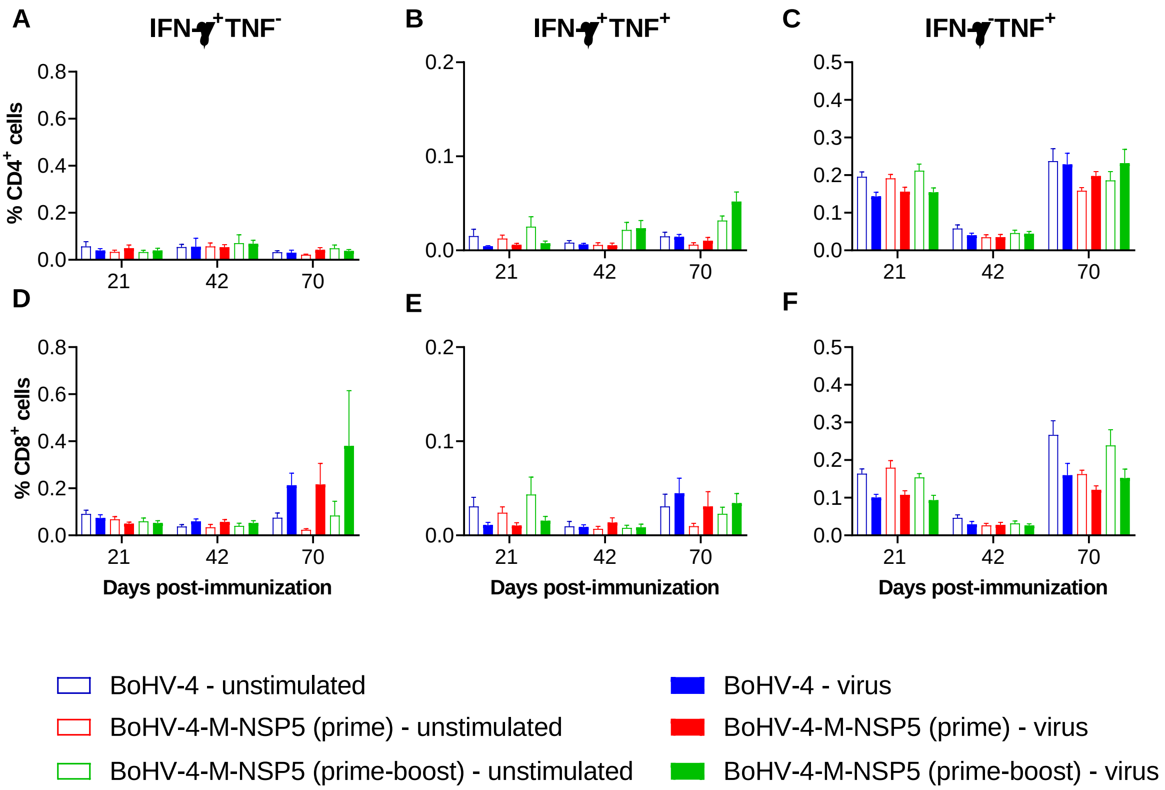

Supplementary Figure 9. Characterization of antigen-specific cytokine responses in the blood of pigs after vaccination with BoHV-4-M-NSP5 and PRRSV-1 challenge. PRRSV-1 M/NSP5 specific cytokine responses were assessed following one (prime) or two (prime-boost) immunizations of pigs with BoHV-4-M-NSP5 and challenge with PRRSV-1 21301/19 (Study 1). Control pigs were immunized twice with the BoHV-4 vector before PRRSV challenge. Responses of previously cryopreserved PBMCs from D21, D42 and D70 were assessed with and without PRRSV-1 21301/19 (virus) stimulation by flow cytometry. Expression of IFN-γ alone (**A**, **D**), IFN-γ and TNF (**B, E**), or TNF alone (**C**, **F**) by CD4 T cells (**A - C**) and CD8 T cells (**D - F**) are shown as mean data ± SD for each vaccine group.

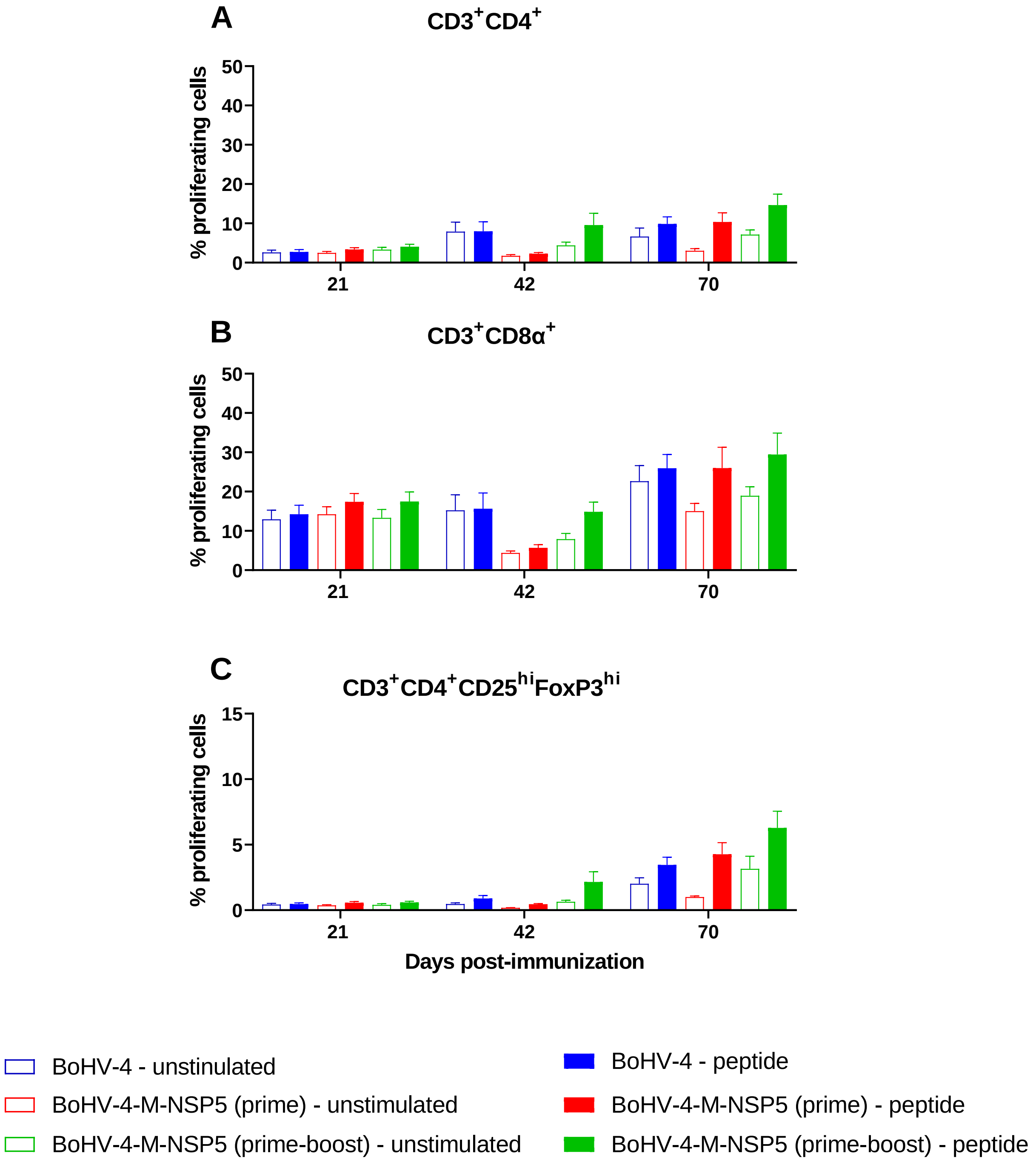

Supplementary Figure 10. Characterization of antigen-specific proliferative responses in the blood of pigs after vaccination with BoHV-4-M-NSP5 and PRRSV-1 challenge. PRRSV-1 M/NSP5 specific proliferative responses were assessed following one (prime) or two (prime-boost) immunizations of pigs with BoHV-4-M-NSP5 and challenge with PRRSV-1 21301/19 (study 1). Control pigs were immunized twice with the BoHV-4 vector before PRRSV challenge. Responses of previously cryopreserved PBMCs from D21, D42 and D70 were assessed with and without peptide stimulation by flow cytometry. The mean % proliferation of CD4 T cells (**A**), CD8 T cells (**B**) and Tregs (**C**) are shown ± SD for each vaccine group.

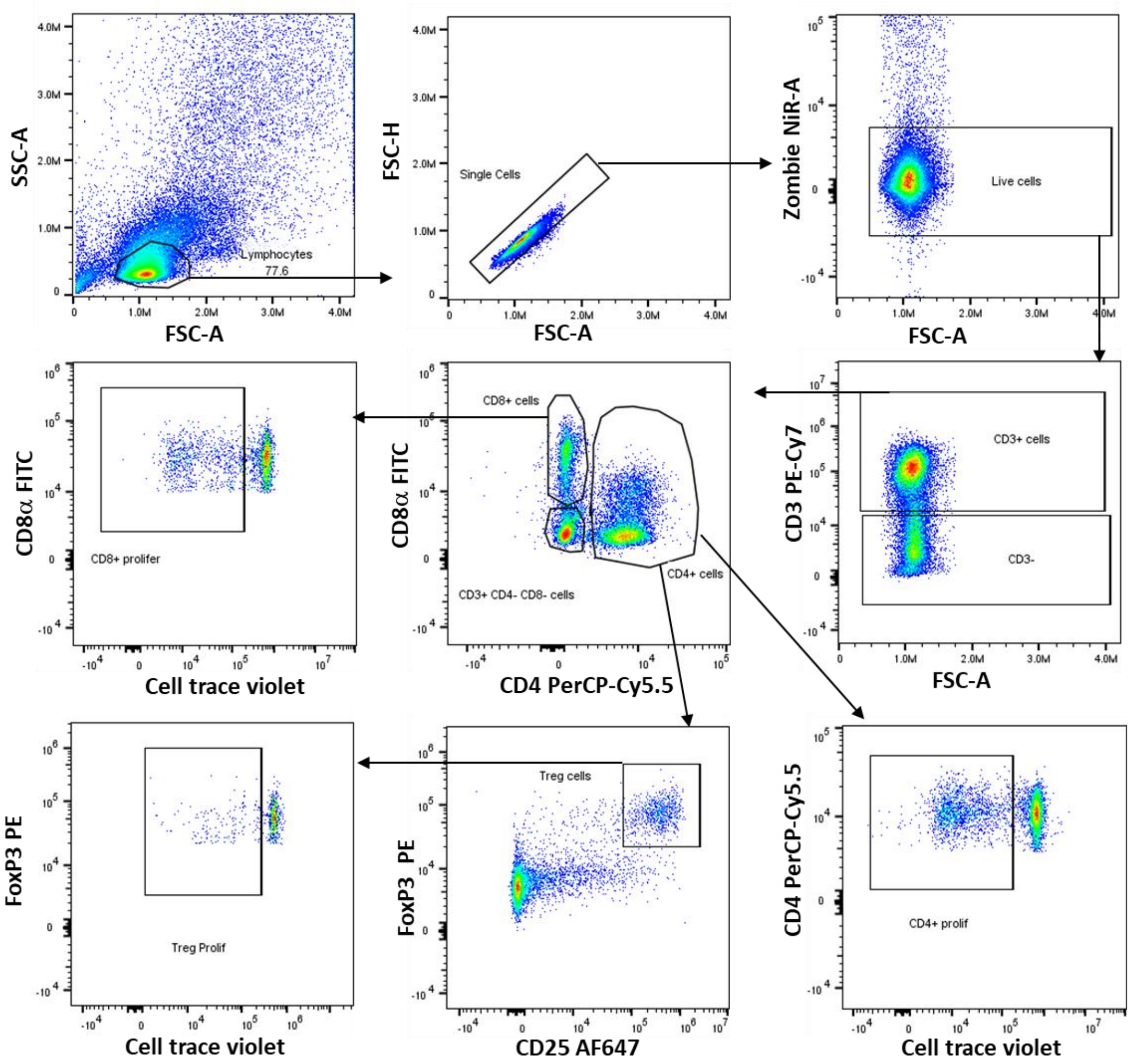

Supplementary Figure 11. Flow cytometric gating strategy for assessment of T cell proliferative responses. Lymphocytes were gated by their characteristic light scattering and then further gated to exclude dead cells. Live lymphocytes were separated into $CD3^+$ T-cells and $CD3^-$ non-T-cells before $CD3^+$ cells were sub-gated for $CD4^+$ T cells, $CD8\alpha^+$ T cells, and Tregs defined at $CD3^+$ $CD4^+$ $CD25^{high}$ $FoxP3^+$. The % of proliferating cells was assessed as those displaying reduced CellTrace™ Violet labelling. Representative dot plots from peptide stimulated PBMC are shown.

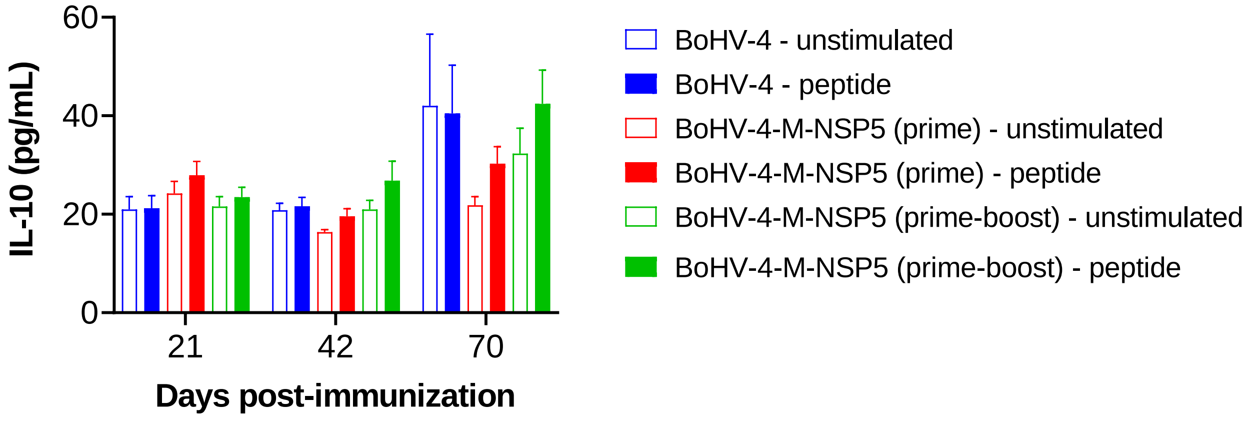

Supplementary Figure 12. Quantification of porcine IL10 in supernatant of peptide stimulated PBMC. PRRSV-1 M/NSP5 specific cytokine responses were assessed following one (prime) or two (prime-boost) immunizations of pigs with BoHV-4-M-NSP5 and challenge with PRRSV-1 21301/19 (study 1). Control pigs were immunized twice with the BoHV-4 vector before PRRSV challenge. IL10 in supernatant of culture of cryopreserved PBMCs from D21, D42 and D70 were assessed following with and without peptide stimulation by ELISA. Mean IL-10 concentrations ± SD are presented for each vaccine group.

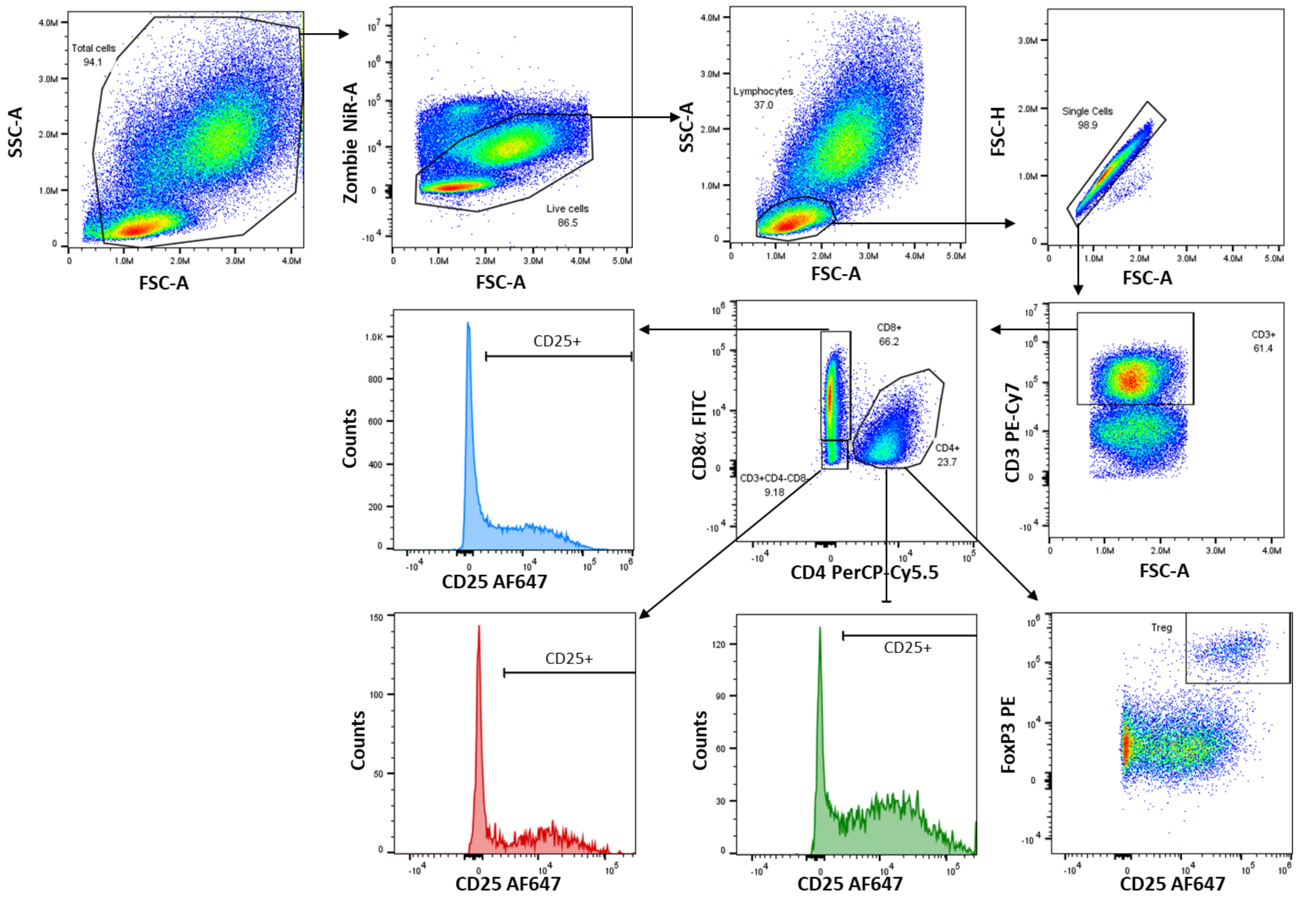

Supplementary Figure 13. Flow cytometric gating strategy for T cell phenotyping of bronchoalveolar lavage cells. Cells were gated by their characteristic light scattering and then further gated to exclude dead cells. Live lymphocytes were separated into $CD3^+$ T-cells and $CD3^-$ non-T-cells before $CD3^+$ cells were sub-gated for $CD4^+$ T cells, $CD8\alpha^{high}$ T cells, non-conventional T cells defined as $CD3^+$ $CD8\alpha^{-/low}$ $CD4^-$ T-cells. The proportion of these cell populations expressing CD25 was then assessed. Tregs were defined at $CD3^+$ $CD4^+$ $CD25^{high}$ $FoxP3^+$.

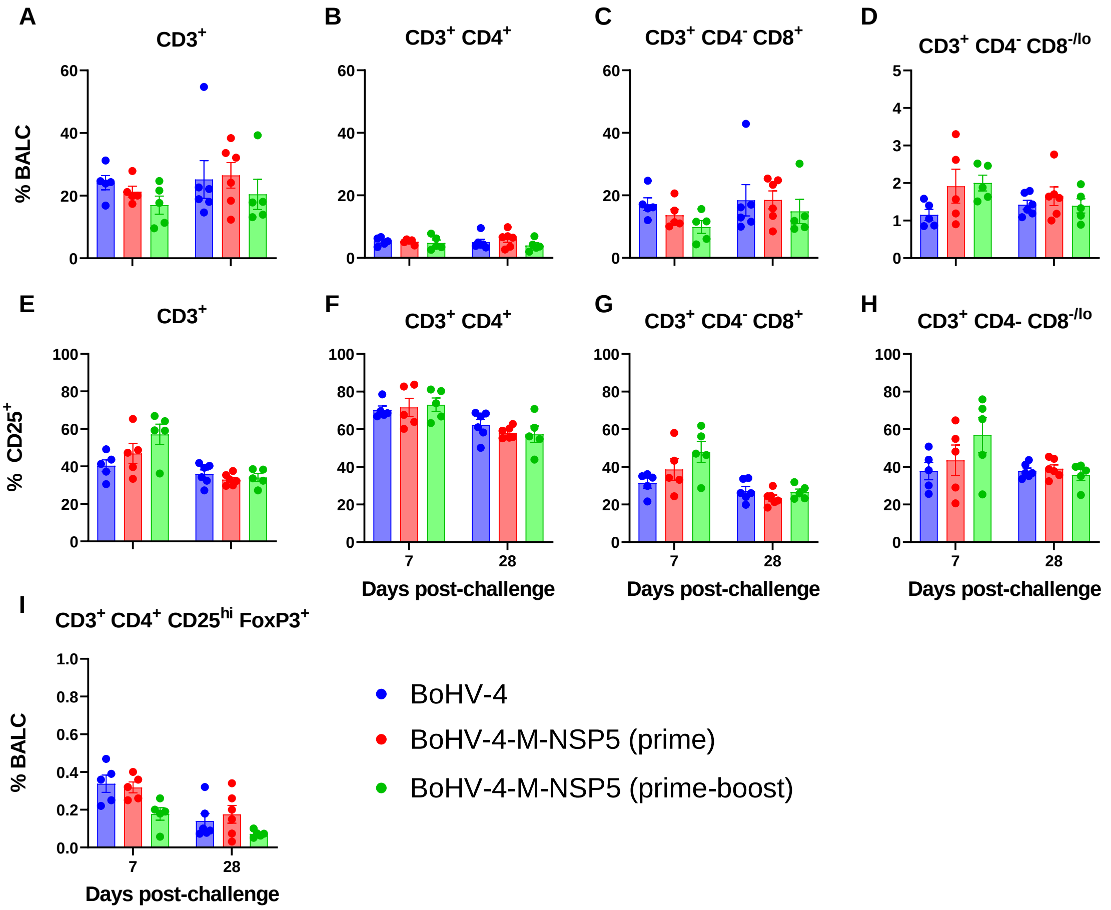

Supplementary Figure 14. Phenotyping of T-cell subsets infiltrating the lungs following BoHV-4-M-NSP5 vaccination and PRRSV-1 challenge. Pigs were immunized once (prime) or twice (prime-boost) with BoHV-4-M-NSP5 or twice with the BoHV-4 vector before challenge with PRRSV-1 21301/19 (study 1). Following euthanasia on 7- and 28-days post-challenge, BALC were isolated. Previously cryopreserved cells were labelled and analyzed by flow cytometry. The proportion of $CD3^+$ T cells (**A**), $CD4^+$ T cells (**B**), $CD8^+$ T cells (**C**), non-conventional T cells (**D**) was assessed. The percentage of these cells expressing the activation marker CD25 was also assessed (**E-H**). The proportion of Tregs in BALC was also determined (**I**). Mean data ± SD for each vaccine group are shown with datapoints representing individual pigs.

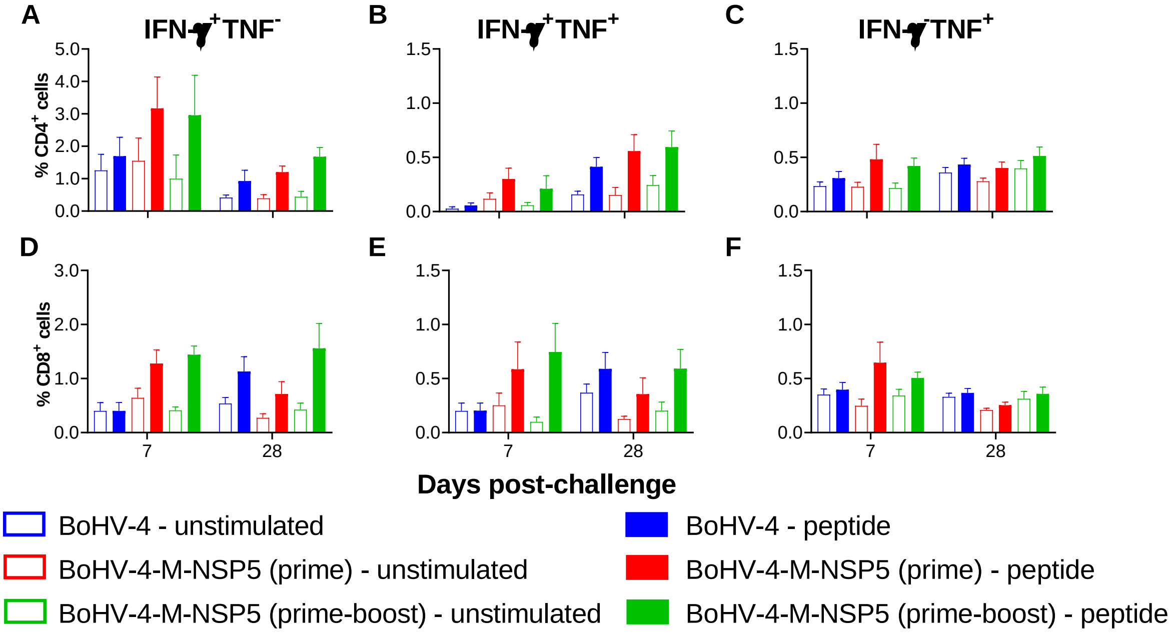

Supplementary Figure 15. Characterization of antigen-specific IFN-γ and TNF responses in the lungs of pigs after vaccination with BoHV-4-M-NSP5 and PRRSV-1 challenge. PRRSV-1 M/NSP5 specific cytokine responses were assessed following one (prime) or two (prime-boost) immunizations of pigs with BoHV-4-M-NSP5 and challenge with PRRSV-1 21301/19 (Study 1). Control pigs were immunized twice with the BoHV-4 vector before PRRSV challenge. Responses of previously cryopreserved BALC from 7- and 28-days post-challenge were assessed with and without peptide stimulation by flow cytometry. Expression of IFN-γ alone (**A**, **D**), IFN-γ and TNF (**B**, **E**), or TNF alone (**C**, **F**,) by CD4 T cells (**A**-**C**) and CD8 T cells (**D**-**F**) are shown as mean data ± SD for each vaccine group.

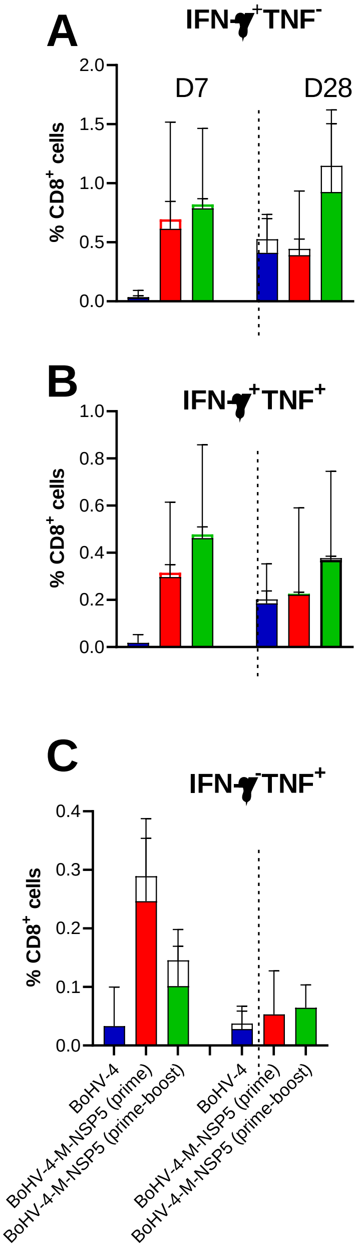

Supplementary Figure 16. Assessment of perforin expression by CD8 T cells after vaccination with BoHV-4-NSP5-M and PRRSV-1 challenge. PRRSV-1 M/NSP5 specific cytokine responses were assessed following one (prime) or two (prime-boost) immunizations of pigs with BoHV-4-NSP5-M and challenge with PRRSV-1 21301/19 (study 1). Control pigs were immunized twice with the BoHV-4 vector before challenge. Responses of previously cryopreserved BALC from 7- and 28-days post-challenge were assessed following peptide stimulation by flow cytometry. Perforin expression (perforin^{+} - empty bars; perforin^{-} - solid bars) by CD8 T cells expressing IFN-γ alone (**A**), IFN-γ and TNF (**B**), or TNF alone (**C**) are shown as mean unstimulated condition corrected data ± SD for each vaccine group.

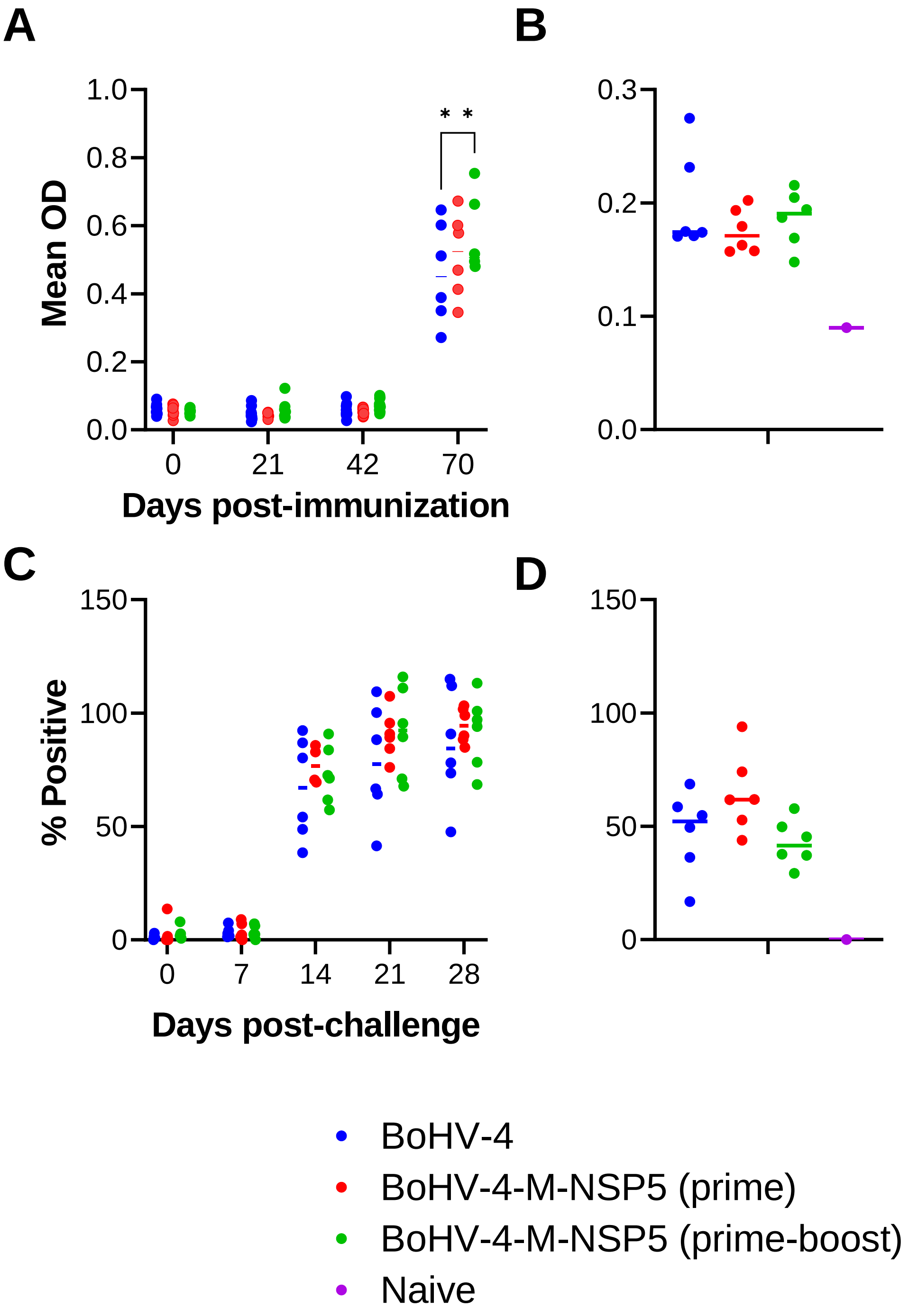

Supplementary Figure 17. Assessment of antibody responses following BoHV-4-M-NSP5 vaccination and PRRSV-1 challenge. Pigs were immunized once (prime) or twice (prime-boost) with BoHV-4-M-NSP5 or twice with the BoHV-4 vector before challenge on D42 with PRRSV-1 21301/19 (study 1). An infected cell lysate was used in ELISA to assess PRRSV-1-specific antibodies in serum (**A**) and BALF (**B**) and a diagnostic ELISA was used to assess PRRSV N protein-specific antibodies in serum (**C**) and BALF (**D**). The results were expressed as OD_{450} or percentage of positivity (PP) according to the formula: PP = (OD_{450} sample − OD_{450} negative control / OD_{450} positive control − OD_{450} negative control) × 100. Datapoints represent individual pigs with the median indicated by a horizontal line. BALF from a PRRSV-naïve pig was included as an additional negative control.

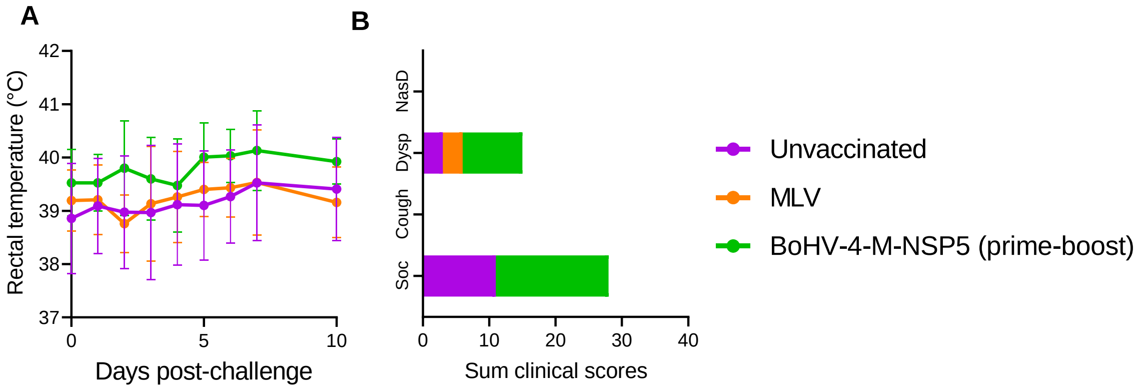

Supplementary Figure 18. Evaluation of the efficacy of a BoHV-4 vectored PRRSV vaccine candidate. The ability BoHV-4-M-NSP5 immunization to confer clinical protection was assessed following challenge infection of pigs with PRRSV-1 LT-3 (Study 2). A negative control group comprised no immunization and a positive control group was immunized with PRRSV-1 MLV. Mean data ± SD for rectal temperatures over the course of challenge (**A**) is shown. The sum of clinical scores observed in the post-challenge period are presented for each group (**B**). Nasal discharge – NasD; dyspnoea – Dysp, and changes in social behavior – Soc.
